# Constant domain polymorphisms influence monoclonal antibody stability and dynamics

**DOI:** 10.1002/pro.4589

**Published:** 2023-02-24

**Authors:** Annmaree K. Warrender, Jolyn Pan, Chris R. Pudney, Vickery L. Arcus, William Kelton

**Affiliations:** ^1^ Te Huataki Waiora School of Health University of Waikato Hamilton New Zealand; ^2^ Te Aka Mātuatua School of Science University of Waikato Hamilton New Zealand; ^3^ Department of Biology and Biochemistry University of Bath Bath UK

**Keywords:** allotypes, antibody stability, constant region alleles, Fc domain, REES

## Abstract

The constant regions of clinical monoclonal antibodies are derived from a select number of allotypes found in IgG subclasses. Despite a long‐term acknowledgment that this diversity may impact both antibody function and developability, there is a lack of data on the stability of variants carrying these mutations. Here, we generated a panel of IgG1, IgG2, and IgG3 antibodies with 32 unique constant region alleles and performed a systematic comparison of stability using red edge excitation shift (REES). This technique exploits the fluorescent properties of tryptophan residues to measure antibody structural dynamics which predict flexibility and the propensity to unfold. Our REES measurements revealed broad stability differences between subclasses with IgG3 possessing the poorest overall stability. Further interrogation of differences between variants within each subclass enabled the high‐resolution profiling of individual allotype stabilities. Crucially, these observed differences were not found to be linked to N297‐linked glycan heterogeneity. Our work demonstrates diverse stabilities (and dynamics) for a range of naturally occurring constant domain alleles and the utility of REES as a method for rapid and sensitive antibody stability profiling, requiring only laboratory spectrophotometry equipment.

## 1 INTRODUCTION

In the 25 years since the first approval of humanized daclizumab with an IgG1 backbone, the diversity of clinically approved antibodies has grown to encompass both IgG2 and IgG4 subclasses (Kaplon et al. 2020). The majority of clinical monoclonal antibodies (mAbs) within each of these subclasses are based on a small number of allotypes encoded by constant region alleles (Jefferis and Lefranc 2009). This is despite the long‐standing recognition that human populations exhibit significant constant region diversity (Vidarsson et al. 2014; Warrender and Kelton 2020). Recent targeted sequencing approaches, and the mining of high‐resolution genome sequences, have further increased known constant region diversity with potential implications for the design of next generation monoclonal drugs (Khatri et al. 2021; Calonga‐Solís et al. 2019).

The constant region of antibodies directly contributes to antibody mediated effector function, immunogenicity, and stability. Antibody engagement of Fc receptors via the constant region mediates potent effector responses, such as antibody‐dependent cellular cytotoxicity (ADCC) and antibody‐dependent cellular phagocytosis (ADCP). Engineered constant region sequences that modulate immune function have been very successful clinically with more than 21 modified mAb drugs approved in the United States to date (The Antibody Society 2022). Moreover, naturally occurring polymorphisms (specifically in the CH2 and hinge domains) have been demonstrated to alter Fc receptor binding and subsequently influence in vitro effector function (de Taeye et al. 2020). Given that this diversity potentially creates new epitopes, it is surprising to find long standing immunogenicity concerns around patient constant region polymorphic disparity with mAb drugs have largely not manifested clinically (Bartelds et al. 2010). There are a few notable exceptions and some reports have highlighted T cell epitope driven mechanisms may account for instances of elevated antidrug antibody levels (Benucci et al. 2018; Stickler et al. 2011).

Alongside low immunogenicity, high stability is fundamental to the developability of reliable mAbs and is inherently linked to dynamic flexibility of the protein structure (Feige et al. 2004; Karshikoff et al. 2015). The relationship between flexibility and stability encompasses a wide range of phenomena (e.g., local unfolding vs loop motions) over a wide range of timescales (ranging from ns to ms). Entropic and enthalpic contributions are unique to each situation and are also heavily influenced by the surrounding solvent. Typically, mAbs exhibiting greater intradomain flexibility have poorer stability than more rigid counterparts although even small environmental perturbations (e.g., temperature increases) are liable to enable conformations predisposed to unfolding or aggregation. Post‐translational glycosylation can further influence antibody stability through interactions with CH2 domain residues (Aoyama et al. 2019; Krapp et al. 2003; Higel et al. 2016). Within the IgG isotype, subclass stability differences are well established, with IgG1 ranked the most stable subclass, followed by IgG2 and IgG4, and finally IgG3 being the least thermally stable and most prone to aggregation (Ito and Tsumoto 2013). Resolving stability differences at the single point mutation level, however, is significantly more challenging and has not been systematically investigated for mutations in antibody constant regions. Traditional methods used to measure protein stability (e.g., differential scanning fluorimetry [DSF], differential scanning calorimetry, or circular dichroism) have been successfully deployed but require the careful preparation of samples to avoid aggregates and the results can be dependent on heating rates and incubation times (Vermeer and Norde 2000; Lin et al. 2015). Alternative nuclear magnetic resonance (Brinson et al. 2019; Arbogast et al. 2015; Nokwe et al. 2014) and hydrogen/deuterium exchange mass spectrometry (More et al. 2018; Majumdar et al. 2015; Houde and Engen 2013) approaches are also excellent for the interrogation of antibody dynamics at high resolution but remain confined to specialist facilities.

**FIGURE 1:**
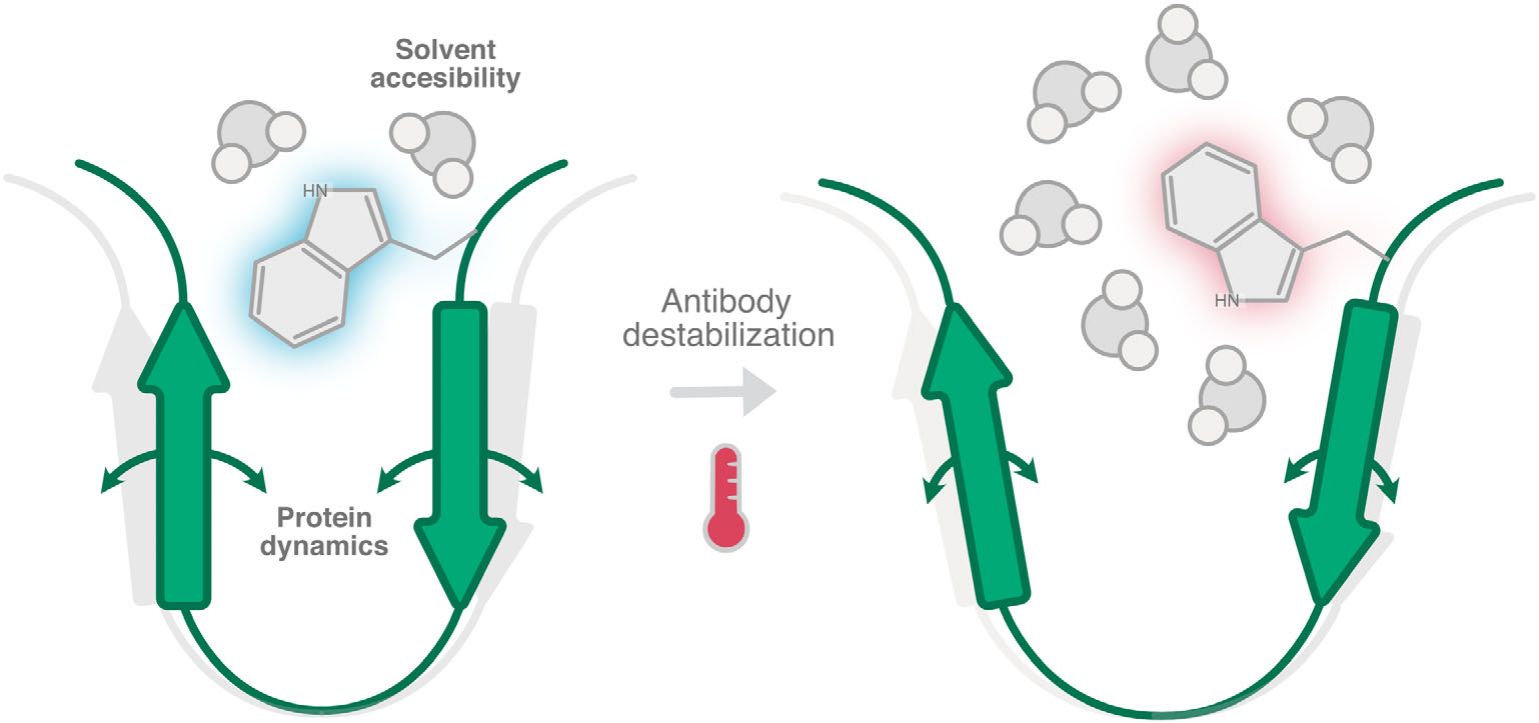
Graphical depiction of the REES effect as determined by the solvent‐accessibility of tryptophan residues. Tryptophan residues in a rigid/folded protein structure typically have limited solvent access causing blue shifted fluorescent emission profiles. Destabilization causes the protein structure to become more flexible, increasing the solvent accessibility to tryptophan and causing a red shifted fluorescent emission profile.

The recent development of red edge excitation shift (REES) spectroscopy for high‐resolution antibody stability analysis offers a rapid and accessible technique using common laboratory equipment (Knight et al. 2020; Thakkar et al. 2012). This highly sensitive approach detects distinct flexibility profiles for structurally identical proteins harboring single point mutation differences (Jones et al. 2017). The underlying principles of REES have been thoroughly discussed in several publications (Catici et al. 2016; Haldar et al. 2011; Kwok et al. 2021). In brief, REES exploits the fluorescent properties of tryptophan (Trp) residues as highly sensitive reporters of the local protein environment (Figure 1). In particular, the fluorescent emission profile of Trp is greatly influenced by the degree of interaction with surrounding water molecules. In stable, folded structures Trp residues are often buried within the structure where solvent molecules are excluded and as a result retain a higher energy of emission with minimal red shift. In contrast, Trp residues in more flexible or partially unfolded proteins can become exposed to solvents, giving rise to a red shift in emission. This creates a unique population of Trp residues that fluoresce at low emission energies regardless of excitation wavelength. Across sequentially lower excitation energies, the emission energy of Trp residues in folded, rigid proteins decreases relative to the excitation wavelength shifting toward the “red‐edge” (The REES phenomenon). REES can therefore be used to interrogate the structural dynamics of antibodies and the changes in flexibility imparted by thermal stress as a means to rapidly quantify mAb stability.

Here, we have used REES to establish relative stability profiles for a panel of 32 IgG1, IgG2, and IgG3 constant region polymorphisms formatted with trastuzumab variable domains. We report broad differences in stability between the subclasses with IgG1 and IgG2 allelic variants displaying greater thermal stability than IgG3 alleles. Importantly, REES also enables high‐resolution discrimination of differences in stability at the allelic/point mutation level. The present work leads us to expect a broader utility of this technique for the high‐throughput screening of potential mAb candidates with enhanced stability.

## 2 RESULTS

### 2.1 Stability profiling of IgG subclasses by red edge excitation shift shows broad differences

**FIGURE 2:**
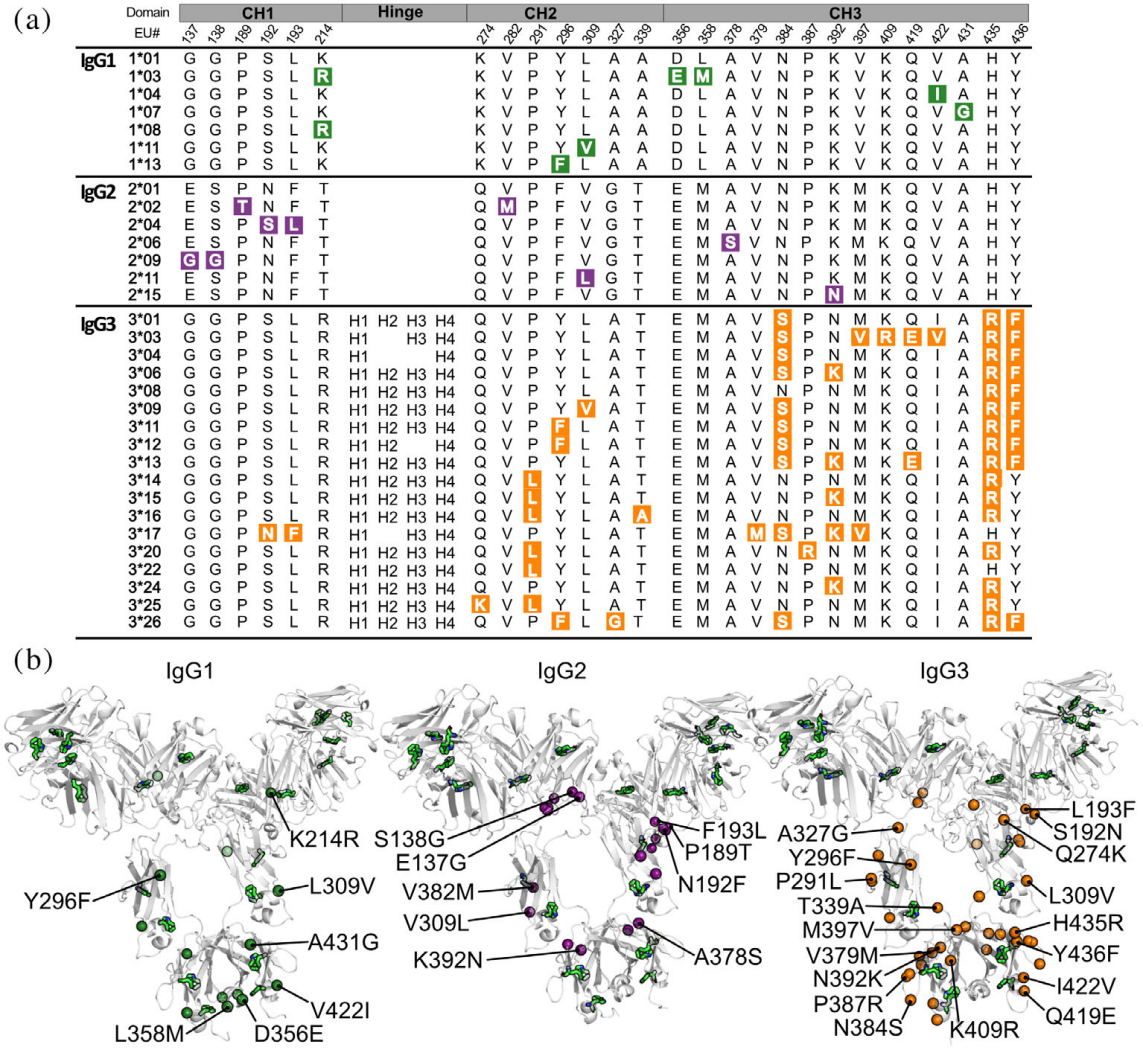
Amino acid polymorphisms in IgG1, IgG2, and IgG3 constant heavy chain alleles. (a) Amino acid position is given based on the EU numbering and divided into the three constant domains and hinge. The hinge sequence is identical for IgG1 and IgG2 alleles. The number of hinge exons differs for IgG3 alleles; H1‐4 denotes what exon is present in each allele. Amino acid polymorphisms are highlighted. (b) Allele polymorphisms are mapped onto representative models of full length IgG1, IgG2, and IgG3 generated using Robetta structural prediction software. Tryptophan residues are distributed throughout the structure and are highlighted in green.

**FIGURE 3:**
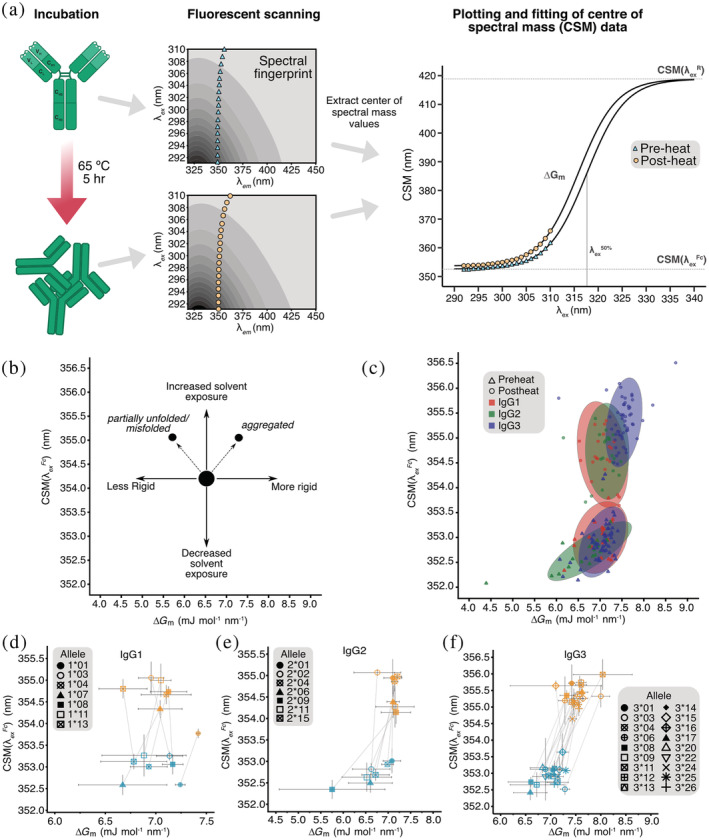
REES analysis of 32 trastuzumab constant region variants. (a) Overview of the REES method: (left) depiction of the state of the antibody measured in each condition, (middle) examples of the emission/excitation matrices of tryptophan residues in the pre‐ and post‐heat samples, contoured from high (black) to low (light gray) fluorescent intensity, (right) model fitting to obtain $\mathrm{CSM}\left(\lambda_{\mathrm{Ex}}^{\mathrm{Fc}}\right)$ and ∆*G*_*m*_ values from the CSM data. (b) Description of the physical state of the antibody given a corresponding directional shift in $\mathrm{CSM}\left(\lambda_{\mathrm{Ex}}^{\mathrm{Fc}}\right)$ and ∆*G*_*m*_ values from the native state. (c) $\mathrm{CSM}\left(\lambda_{\mathrm{Ex}}^{\mathrm{Fc}}\right)$ verses ∆*G*_*m*_ for native (triangle) and heat stressed (circle) antibodies from all subclasses; IgG1 (red), IgG2 (green), and IgG3 (blue). Datapoints are of each triplicate measurement for each antibody sample. The colored ellipses show the clustering of 95% of the data for a given subclass and condition. (d–f) $\mathrm{CSM}\left(\lambda_{\mathrm{Ex}}^{\mathrm{Fc}}\right)$ verses ∆*G*_*m*_ plots of antibodies in subclasses IgG1‐3, respectively. For each allele, the native antibody state (blue) is connected to the heat stressed state (orange).

Thirty‐two trastuzumab variants each possessing a unique constant region allele (Figure 2a,b) containing equal numbers of tryptophan (Trp) residues were isolated at high purity following expression (Figure S1). Homology modeling of IgG1 allelic variants, using template based Robetta software, predicted only minor structural differences despite the known influences of these mutations on antibody function (Figure S2 and Table S2). We therefore concluded the contributions of allelic variation to variant structure and stability would be better captured by REES analysis of dynamic antibody flexibility. Fluorescent spectra were obtained before and after heating in a pH 8 buffer designed to induce destabilization over a timescale amenable to analysis in a single day (Cheng et al. 2012) (Figure 3a). A recently developed thermodynamic model for REES was then fitted to the fluorescent data to extract values for $\mathrm{CSM}\left(\lambda_{\mathrm{Ex}}^{\mathrm{Fc}}\right)$ and ∆*G*_*m*_ that allow for biologically relevant insights to be drawn from the data (Figures 3b and S3) (Kwok et al. 2021). Specifically, $\mathrm{CSM}\left(\lambda_{\mathrm{Ex}}^{\mathrm{Fc}}\right)$ provides information about the degree of tryptophan residue exposure to the surrounding solvent (a measure of protein foldedness) and ∆*G*_*m*_ describes the rigidity of the protein, which is linked to the aggregation state.

Values of $\mathrm{CSM}\left(\lambda_{\mathrm{Ex}}^{\mathrm{Fc}}\right)$ and Δ*G*_*m*_ were calculated for the pre‐heated samples to set a baseline for initial stability characteristics of each individual antibody. Subsequent increases in $\mathrm{CSM}\left(\lambda_{\mathrm{Ex}}^{\mathrm{Fc}}\right)$ following heating are indicative of increased Trp exposure to the surrounding buffer and were observed for all variants tested. When assessed in conjunction with values of ∆*G*_*m*_, insights into antibody unfolding dynamics and aggregation propensity are obtained. Associated decreases in ∆*G*_*m*_ suggest greater flexibility indicating partial unfolding whereas increases in ∆*G*_*m*_ indicate loss of flexibility due to aggregation.

The value of the center of spectral mass (CSM) of the completely relaxed state of emission ($\mathrm{CSM}\left(\lambda_{\mathrm{Ex}}^{R}\right)$) was obtained by global fitting of data collected between $\lambda_{\mathrm{Ex}}$ = 292–310 nm (Kwok et al. 2021) and was 418.9 ± 3 nm for our trastuzumab variants. Individual variant fits all displayed regression *p*‐values of less than 0.05 (Figure S3). Within individual subclasses, IgG1 and IgG2 variants shared similar structural dynamics with post‐heat $\mathrm{CSM}\left(\lambda_{\mathrm{Ex}}^{\mathrm{Fc}}\right)$ values clustered around 354.6 ± 0.5 and 354.7 ± 0.5 nm, respectively, while IgG3 variants exhibited larger post‐heat $\mathrm{CSM}\left(\lambda_{\mathrm{Ex}}^{\mathrm{Fc}}\right)$ values centered around 355.3 ± 0.4 nm (*p* < 0.001) (Figure 3c). This trend was likewise consistent for post‐heat ∆*G*_*m*_ values; IgG1 and IgG2 variants had average post‐heat ∆*G*_*m*_ values of 7.1 ± 0.3 and 7.1 ± 0.3 mJ mol^−1^ nm^−1^, significantly lower than ∆*G*_*m*_ = 7.5 ± 0.3 mJ mol^−1^ nm^−1^ (*p* < 0.001) for IgG3 variants.

Subclass stability rankings were supported by DSF for a subset of the antibody alleles measured. DSF curves for each variant showed two unfolding transitions characteristic of IgG antibodies (Figure S4) (Ito and Tsumoto 2013; Richardson et al. 2019; Damelang et al. 2019). On average, the first transition (*T*_m_1) of IgG3 variants was 66.3 ± 1.5°C, significantly lower than 69.7 ± 0.5 and 69 ± 0.5°C for IgG1 and IgG2 variants respectively (*p* < 0.001, Table 1). Similarly, the second transition (*T*_m_2) for IgG3 variations (76.8 ± 0.7°C) was significantly lower (*p* = 0.016) than IgG1 *T*_m_2 (79.7 ± 0.1°C). The average *T*_m_2 values for IgG2 and IgG3 variants fell within the margin of error. Within each subclass, only differences in *T*_m_1 values were distinguishable between allelic variants. IgG1 variant 1*01 had the highest *T*_m_1 of 70.1 ± 0.2°C, significantly higher than the *T*_m_1 of 69.3 ± 0.1°C for 1*11 (*p* = 0.0079). Likewise, 3*17 exhibited the highest *T*_m_1 of 68.5 ± 0.1°C, significantly higher (*p* < 0.01) than all other IgG3 variants tested. There were no discernable differences between the IgG2 variants tested, nor for three of the IgG3 variants (3*03, 3*09, and 3*16).

**TABLE 1:** Differential scanning fluorimetry measurements of eight unique variants, using SYPRO orange dye, reported as the average and standard deviation of triplicate measurements.

Allele	Tm1 (avg)	Tm1 (stdev)	Tm2 (avg)	Tm2 (stdev)
1*01	70.1	0.2	79.7	0.1
1*11	69.3	0.1	79.7	0.1
2*04	69.3	0.3	74.9	3.4
2*09	68.6	1.2	79.6	0.0
3*03	65.4	0.2	75.8	2.2
3*09	65.5	0.2	76.7	1.0
3*16	65.9	1.0	77.3	0.5
3*17	68.5	0.1	77.3	1.3
IgG1 average	69.7	0.5	79.7	0.1
IgG2 average	69.0	0.5	77.2	3.4
IgG3 average^#^	66.3	1.5	76.8	0.7

### 2.2 Red edge excitation shift analysis reveals individual allelic stability fingerprints

#### 2.2.1 IgG1 allelic variation

**FIGURE 4:**
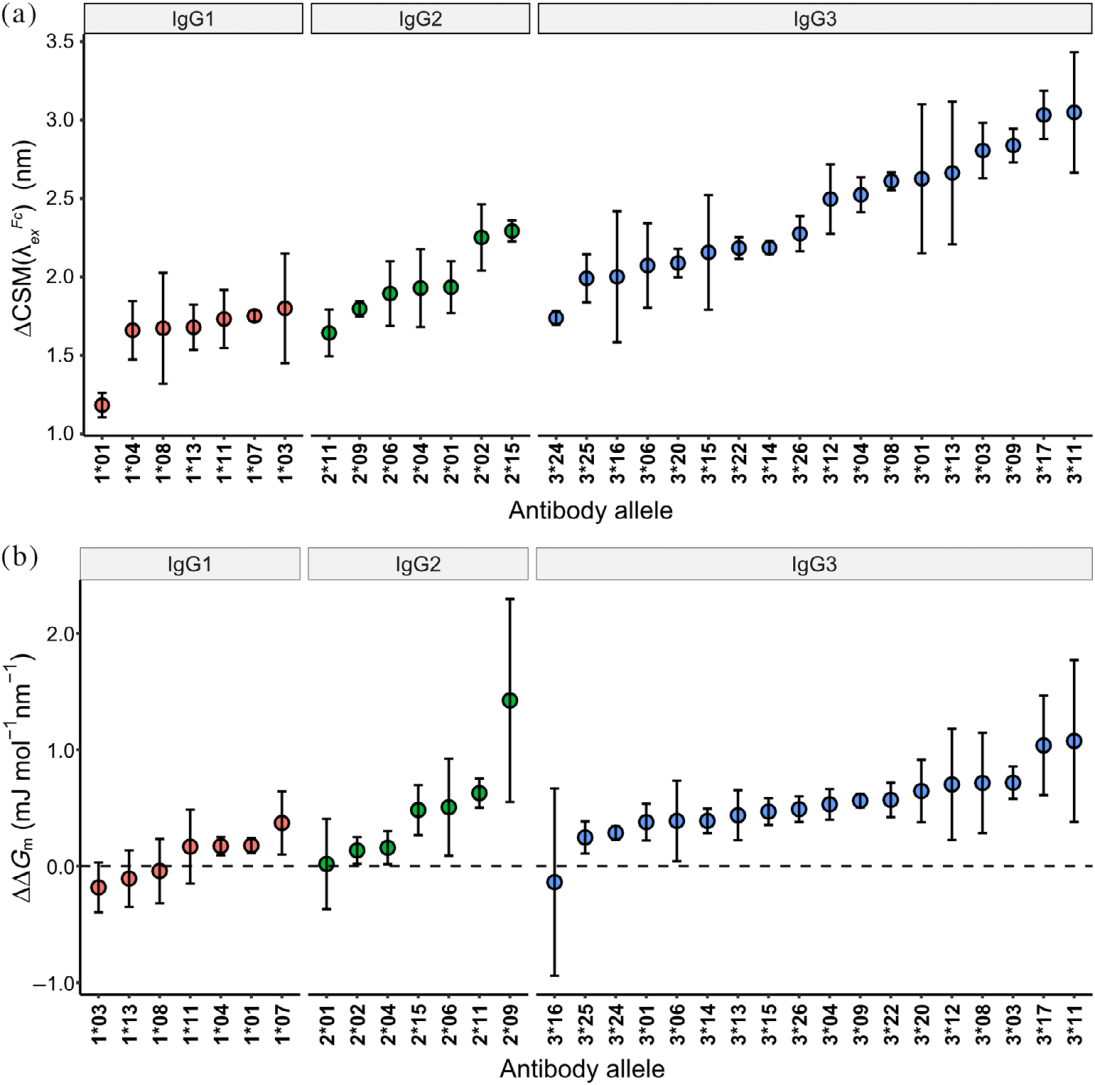
The magnitude of change in $\mathrm{CSM}\left(\lambda_{\mathrm{Ex}}^{\mathrm{Fc}}\right)$ (a) and ∆*G*_*m*_ (b) values for each allele after heating. Data is presented in ascending order of change within each subclass.

Having identified trends in stability at the subclass level, we profiled the relative stabilities of individual antibody constant region alleles. Within the panel of IgG1 variants, significant differences were evident in the pre‐heated $\mathrm{CSM}\left(\lambda_{\mathrm{Ex}}^{\mathrm{Fc}}\right)$ values indicating differing levels of Trp exposure in the native state (Figure 3d). Variants 1*07 and 1*01 had respective pre‐heated $\mathrm{CSM}\left(\lambda_{\mathrm{Ex}}^{\mathrm{Fc}}\right)$ values of 352.6 ± 0.5 and 352.6 ± 0 nm, the lowest of all the IgG1 variants. These values were distinct from variants 1*03 and 1*11 which had respective $\mathrm{CSM}\left(\lambda_{\mathrm{Ex}}^{\mathrm{Fc}}\right)$ values of 353.3 ± 0.1 and 353.3 ± 0.5 nm (*p* < 0.05). However, ∆*G*_*m*_ was not significantly different between any of these variants. After heating, 1*01 had the smallest increase in $\mathrm{CSM}\left(\lambda_{\mathrm{Ex}}^{\mathrm{Fc}}\right)$ of all antibody alleles ($\text{ΔCSM}\left(\lambda_{\mathrm{Ex}}^{\mathrm{Fc}}\right)$ = 1.2 ± 0.1 nm) and only a slight increase in ∆*G*_*m*_ (Δ∆*G*_*m*_ = 0.2 ± 0.1 mJ mol^−1^ nm^−1^) (Figure 4). Together these values suggest a high tolerance to temperature induced unfolding and aggregation and indicate 1*01 is the most stable of the tested alleles. By comparison, 1*03 had a much larger increase in $\mathrm{CSM}\left(\lambda_{\mathrm{Ex}}^{\mathrm{Fc}}\right)$ of 1.8 ± 0.4 nm (*p* = 0.051) and a decrease in ∆*G*_*m*_ of 0.2 ± 0.2 mJ mol^−1^ nm^−1^ indicating greater solvent exposure of Trp after heating likely due to unfolding.

#### 2.2.2 IgG2 allelic variation

Compared to IgG1, IgG2 variants exhibited more variation in the measured REES profiles (Figures 3e and 4). The native IgG2 variants all displayed similar $\mathrm{CSM}\left(\lambda_{\mathrm{Ex}}^{\mathrm{Fc}}\right)$ and ∆*G*_*m*_ profiles with the exception of 2*09 which had a pre‐heated $\mathrm{CSM}\left(\lambda_{\mathrm{Ex}}^{\mathrm{Fc}}\right)$ of 352.3 ± 0.2 nm that was significantly lower than 2*01 ($\mathrm{CSM}\left(\lambda_{\mathrm{Ex}}^{\mathrm{Fc}}\right)$ = 353.0 ± 0.3 nm, *p* = 0.034). Despite similar pre‐heat REES profiles, differences in the magnitude of change for $\;\mathrm{CSM}\left(\lambda_{\mathrm{Ex}}^{\mathrm{Fc}}\right)$ suggested unique thermostability fingerprints for IgG2 variants. After heating, 2*11 had the smallest $\text{ΔCSM}\left(\lambda_{\mathrm{Ex}}^{\mathrm{Fc}}\right)$ of 1.6 ± 0.2 nm relative to 2*02 ($\text{ΔCSM}\left(\lambda_{\mathrm{Ex}}^{\mathrm{Fc}}\right)$ = 2.3 ± 0.2 nm, *p* = 0.0091) and 2*15 variants ($\text{ΔCSM}\left(\lambda_{\mathrm{Ex}}^{\mathrm{Fc}}\right)$ = 2.3 ± 0.1 nm, *p* = 0.0053). However, there were no significant differences between the changes in ∆*G*_*m*_ for these variants. We also note 2*09 and 2*01 variants showed similar increases in $\mathrm{CSM}\left(\lambda_{\mathrm{Ex}}^{\mathrm{Fc}}\right)$ of 1.8 ± 0.1 and 2 ± 0.2 nm, respectively, despite differences in pre‐heat values. The increase in ∆*G*_*m*_ for 2*09 after heating was 1.4 ± 0.9 mJ mol^−1^ nm^−1^ which was significantly larger than 2*01 (Δ∆*G*_*m*_ = 0.02 ± 0.4 mJ mol^−1^ nm^−1^, *p* = 0.012), 2*02 (Δ∆*G*_*m*_ = 0.1 ± 0.1 mJ mol^−1^ nm^−1^, *p* = 0.023) and 2*04 (Δ∆*G*_*m*_ = 0.2 ± 0.1 mJ mol^−1^ nm^−1^, *p* = 0.026).

#### 2.2.3 IgG3 allelic variation

IgG3 variants in the native, pre‐heated state exhibited similar $\mathrm{CSM}\left(\lambda_{\mathrm{Ex}}^{\mathrm{Fc}}\right)$ and ∆*G*_*m*_ values to each other with a few exceptions (Figure 3f). Variant 3*16 had the highest pre‐heated $\mathrm{CSM}\left(\lambda_{\mathrm{Ex}}^{\mathrm{Fc}}\right)$ value of 353.6 ± 0.6 nm, illustrating a state of Trp solvent exposure distinct from variants 3*17 and 3*03 ($\mathrm{CSM}\left(\lambda_{\mathrm{Ex}}^{\mathrm{Fc}}\right)$ = 352.4 ± 0.2, *p* = 0.0075 and 352.5 ± 0.2 nm, *p* = 0.021, respectively). The structural rigidity represented by ∆*G*_*m*_, however, appeared to be within the margin of error between these alleles. After heating, the magnitude of change for $\mathrm{CSM}\left(\lambda_{\mathrm{Ex}}^{\mathrm{Fc}}\right)$ and ∆*G*_*m*_ differed between variants (Figure 4). IgG3*24 showed the smallest shift in $\mathrm{CSM}\left(\lambda_{\mathrm{Ex}}^{\mathrm{Fc}}\right)$, increasing by only 1.7 ± 0.04 nm to 352.9 ± 0.2 nm. This was significantly smaller than eight other IgG3 variants (3*01, *03, *04, *08, *09, *11, *13, and *17, *p* < 0.05). Variants 3*11 and 3*17 appeared to be the least stable, with values of $\mathrm{CSM}\left(\lambda_{\mathrm{Ex}}^{\mathrm{Fc}}\right)$ increasing by 3.1 ± 0.4 and 3.0 ± 0.2 nm, respectively. This increase was significantly greater than eight other IgG3 alleles (3*06, *14, *15, *16, *20, *22, *24 and *25, *p* < 0.05). The values of ∆*G*_*m*_ for 3*11 and 3*17 increased by 1.1 ± 0.7 and 1.0 ± 0.4 mJ mol^−1^ nm^−1^, respectively, distinctly larger increases than the most stable IgG3 variant, 3*16 (Δ∆*G*_*m*_ = −0.1 ± 0.8 mJ mol^−1^ nm^−1^, *p* = 0.011 and 0.016, respectively).

### 2.3 Specific amino acid substitutions explain differences in red edge excitation shift profiles

**FIGURE 5:**
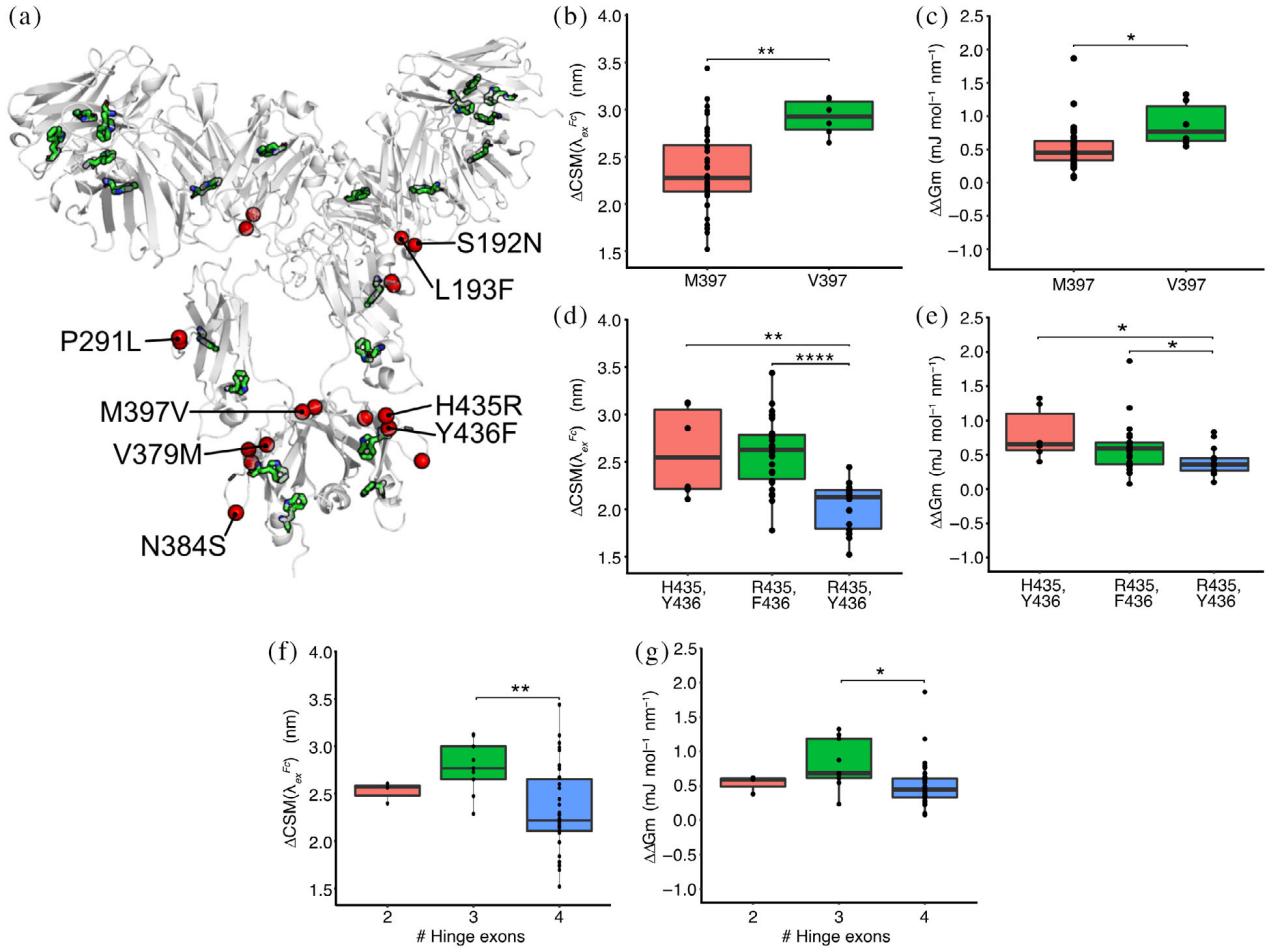
The effect of allelic variation on stability. Data is for IgG3 alleles only. (a) Structural map of amino acid substitutions found in IgG3 alleles (red spheres) that significantly correlated to stability. The location of Trp residues is also shown (green sticks). (b–g) The magnitude of change in $\mathrm{CSM}\left(\lambda_{\mathrm{Ex}}^{\mathrm{Fc}}\right)$ and ∆*G*_*m*_ for amino acid variants at position 397 (b, c), positions 435 and 436 combined (d, e) and hinge length variants (f, g) as measured using REES. Statistical significance was tested by one‐way ANOVA with Tukey post hoc multiple comparison testing. * *p* < 0.05, ** *p* < 0.01, *** *p* < 0.001, **** *p* < 0.0001.

Specific amino acid mutations are known to play a direct role in antibody domain stabilization (Teplyakov et al. 2013; Saito et al. 2019). We therefore undertook a systematic analysis to correlate REES stability metrics with amino acid substitutions present in the IgG3 variant panel (possessing the greatest number of individual alleles with shared mutations) (Figure 2a). Polymorphisms at positions 192, 193, 291, 379, 384, and 397 (Figure 5a) were significantly correlated to changes in both $\mathrm{CSM}\left(\lambda_{\mathrm{Ex}}^{\mathrm{Fc}}\right)$ and ∆*G*_*m*_, (Figures 5b,c and S5). Of particular interest is residue 397 as it is located at the interface between the CH3 domains (Figure S6), suggesting a possible role in stabilizing the antibody structure (Rispens et al. 2014). IgG3 alleles harbor a methionine (Met) or valine (Val) at this position. Variants with Met‐397 had an average increase in $\mathrm{CSM}\left(\lambda_{\mathrm{Ex}}^{\mathrm{Fc}}\right)$ of 2.3 ± 0.4 nm, a smaller increase compared to variants with Val‐397 ($∆\mathrm{CSM}\left(\lambda_{\mathrm{Ex}}^{\mathrm{Fc}}\right)$ = 2.9 ± 0.2 nm, *p* = 0.00136). Met‐397 variants also had a smaller increase in Δ∆*G*_*m*_ of 0.5 ± 0.4 mJ mol^−1^ nm^−1^ compared to 0.9 ± 0.3 mJ mol^−1^ nm^−1^, *p* = 0.02 (Figure 5b,c).

We further interrogated the influence of combinations of allelic mutations. This analysis revealed the influence of amino acid mutations at positions 435 and 436 of the IgG3 CH3 domain. Three combinations of alleles are observed within the IgG3 subclass; His‐435 and Tyr‐436, Arg‐435 and Tyr‐436, or Arg‐435 and Phe‐436. Upon exposure to heat, Arg‐435/Tyr‐436 variants exhibited the smallest change in both $\mathrm{CSM}\left(\lambda_{\mathrm{Ex}}^{\mathrm{Fc}}\right)$ (2.0 ± 0.3 nm) and ∆*G*_*m*_ (0.3 ± 0.4 J mol^−1^ nm^−1^) compared to His‐435/Tyr‐436 ($\text{ΔCSM}\left(\lambda_{\mathrm{Ex}}^{\mathrm{Fc}}\right)$ = 2.6 ± 0.5 nm, *p* = 0.0018, ∆∆*G*_*m*_ = 0.8 ± 0.4 J mol^−1^ nm^−1^, *p* = 0.018) and Arg‐435/Phe‐436 ($\text{ΔCSM}\left(\lambda_{\mathrm{Ex}}^{\mathrm{Fc}}\right)$ = 2.6 ± 0.4 nm, *p* < 0.001, ∆∆*G*_*m*_ = 0.6 ± 0.4 J mol^−1^ nm^−1^, *p* = 0.032) variants (Figure 5d,e). These small differences in REES parameters suggest the Arg‐435/Tyr‐436 variant is significantly more resistant to heat induced aggregation than either His‐435/Tyr‐436 and Arg‐435/Phe‐436 variants, indicating these mutations likely confer greater stability.

Finally, we investigated the influence of IgG3 hinge length on stability. The hinge length of IgG3 alleles varies significantly depending on the number of hinge exons present (H1‐4) (Figure 2a). Differences in stability were evident between variants with three hinge exons and those with four. Four‐exon variants had significantly smaller changes in REES parameters ($\text{ΔCSM}\left(\lambda_{\mathrm{Ex}}^{\mathrm{Fc}}\right)$ = 2.3 ± 0.4 nm and Δ∆*G*_*m*_ = 0.5 ± 0.4 mJ mol^−1^ nm^−1^) compared to variants with three exons ($\text{ΔCSM}\left(\lambda_{\mathrm{Ex}}^{\mathrm{Fc}}\right)$ = 2.9 ± 0.3 nm, *p* = 0.0084 and Δ∆*G*_*m*_ = 0.8 ± 0.4 mJ mol^−1^ nm^−1^, *p* = 0.037) (Figure 5f,g). Variants with two hinge exons reported values in between ($\text{ΔCSM}\left(\lambda_{\mathrm{Ex}}^{\mathrm{Fc}}\right)$ = 2.5 ± 0.1 nm and Δ∆*G*_*m*_ = 0.5 ± 0.1 mJ mol^−1^ nm^−1^).

### 2.4 Antibody variants share heterogeneous glycosylation patterns regardless of sequence

**FIGURE 6:**
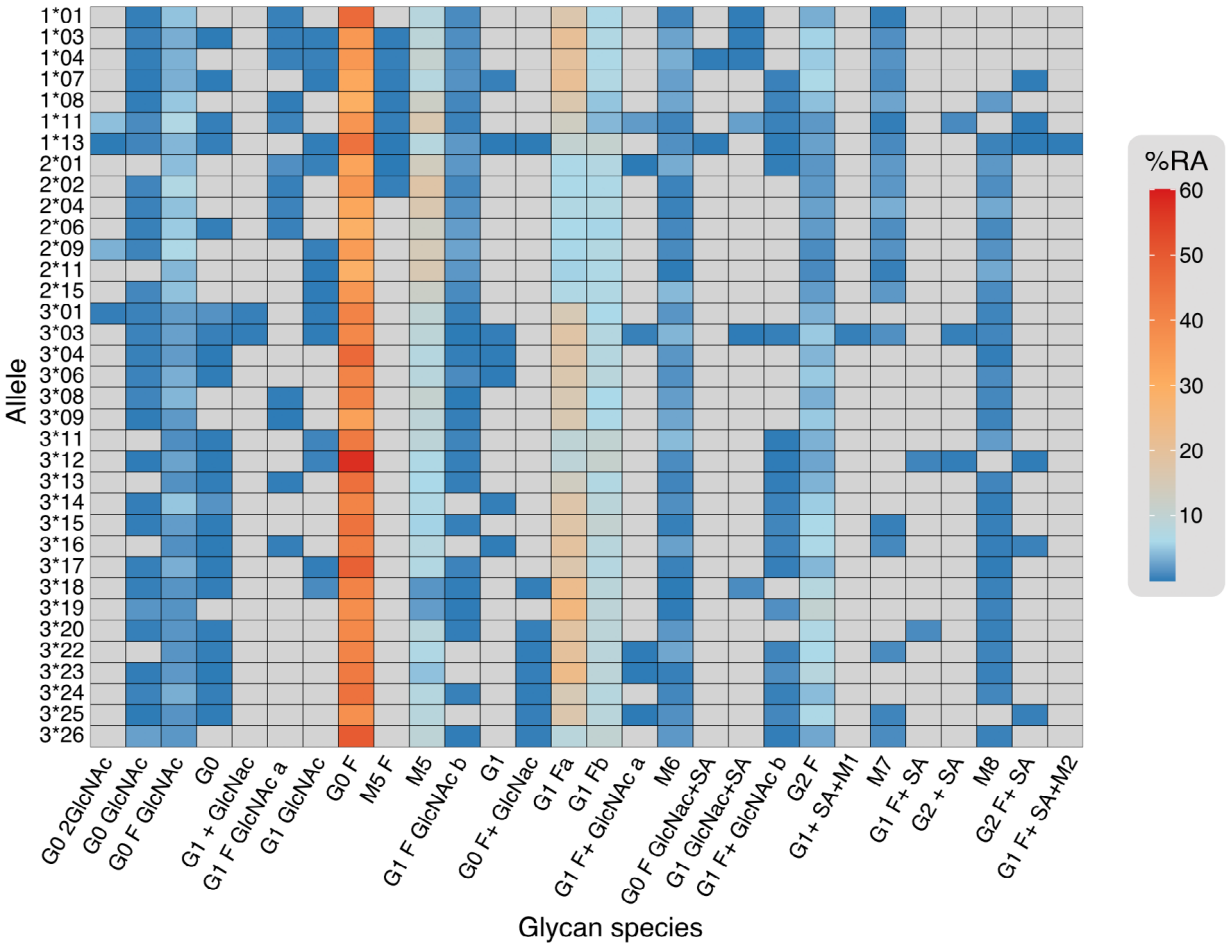
Heatmap showing the relative abundance (RA) of each glycoform appended to each unique antibody. The color scale (right) shows the relative abundance as a percentage. Gray cells are present where the glycoform was not detected or had less than 0.05% RA. Actual RA percentages are shown in Table S3.

To confirm that observed stability differences were a result of allelic point mutations and not glycan structural differences, we performed high‐resolution glycosylation analysis for each variant. Mass spectrometry revealed individual variants from all subclasses shared largely similar heterogeneous glycan profiles consistent with those reported for clinical trastuzumab antibodies (Figure S7a) (Segu et al. 2020). The glycan profiles were dominated by core‐fucosylation (fucose attached to the main chain of the glycan structure, e.g., G0 F, G1 F) and varying degrees of terminal galactosylation (galactose appended to the antennae of the glycan, e.g., G0, G1, and G2) (Figure 6 and Table S3). In some cases, G0 F structures were present at up to 57.5% of the total glycan content. We also observed a varying degree of mannosylation (e.g., M5, M6, and M7) with some bias toward higher levels in IgG2 antibodies. The five most prevalent glycoforms across all of the antibody alleles, in order of decreasing abundance, were G0 F > G1 Fa ≥ M5 > G1 Fb > G2 F. G1 F is present in two isoforms that differ in the position of the terminal galactose residue (G1 Fa [Man α1‐6] and G1 Fb [Man α1‐3]). We detected differences in the ratio of G1 Fa: G1 Fb isoforms between antibody variants. Variants with Phe‐296 (all IgG2 variants, 1*13, 3*11, 3*12, and 3*26) had an equal abundance of each G1 F isoform whereas all variants with Tyr‐296 had three to four times more G1 Fa compared to G1 Fb. Despite variation in the abundance of G1 F isoforms and minor variation between other glycan species, no significant correlations were detected between glycosylation patterns and the $\text{ΔCSM}\left(\lambda_{\mathrm{Ex}}^{\mathrm{Fc}}\right)$ and ∆∆*G*_*m*_ values measured by REES (Figure S7b).

## 3 DISCUSSION

Recent high‐resolution interrogation of the constant region locus has revealed significant population‐linked allelic diversity within antibody subclasses. As therapeutic mAb designs are increasingly exploring alternative antibody constant region scaffolds, we report here the relative thermal stability of 32 constant region alleles from IgG1, IgG2, and IgG3 subclasses, which is one key determinant of mAb developability. We used REES spectroscopy to provide information about antibody structural dynamics and found broad differences between each antibody subclass that were supported by DSF measurements. In addition, we describe striking differences between certain individual alleles of the same subclass (Figure 3d–f). These differences were not contingent on glycan pattern differences between each antibody. Further analysis of our REES data resolved the significant contribution of specific groups of point mutations to overall stability including at positions 435/436 which are known to contribute to antibody half‐life.

While REES has been used for broad profiling of clinical mAb stabilities (Knight et al. 2020), there have been limited reports on the resolution of differences at the point mutation level. Other techniques offer potentially higher resolution for stability analysis but few are as accessible and high‐throughput as REES. Despite these advantages, REES remains unsuitable for the comparative analysis of proteins with different tryptophan content. In this study, three IgG3 allelic variants were excluded from the analysis due to extra tryptophan residues arising from an Arg > Trp mutation in the CH2 domains. Care should also be taken to ensure high quality spectra are obtained from buffer blank samples to eliminate confounding Raman scattering effects.

Nonetheless, the results of our REES analysis show clear differences in stability between the subclasses, with IgG3 exhibiting poorer stability than IgG1 and IgG2 isotypes in alignment with other studies (Garber and Demarest 2007). The REES data suggests IgG3 antibodies have greater hydrophobic residue exposure to the surrounding aqueous environment and a loss of structural flexibility following heat stress, indicative of aggregation. Our findings confirm existing concerns around the stability of IgG3 antibodies, which alongside poor half‐life, have hindered their utility as therapeutic mAbs despite offering strong neutralization activity against pathogens and eliciting potent ADCP and ADCC effector functions (Tay et al. 2016; Bruhns et al. 2009; Richardson et al. 2019; Damelang et al. 2019). Variants belonging to the IgG1 and IgG2 subclasses had relatively similar stability profiles and showed greater resistance to aggregation compared to IgG3. The development of antibody products with reduced effector function, such as where neutralization is desired, has led to a rise in the prevalence of IgG2 mAbs (Andualem et al. 2020; Doñate et al. 2016). IgG4 mAbs are also increasing in clinical popularity but this subclass was not tested in this study due to a lack of complete allelic sequences in the IMGT database. As the number of alleles continues to increase, future REES studies will help to illuminate the relative stability and profile the unique heavy chain exchange dynamics of this isotype.

Despite the relatively large uncertainty in certain variant measurements, REES nonetheless provided sufficient resolution to allow us to rapidly identify differences in stability between individual allelic variants, particularly where no discernable differences were detected by DSF. Crucially, these differences were not due to glycan heterogeneity between alleles and glycan species proportions closely matched those previously reported (Segu et al. 2020; Raju and Jordan 2012; Chen and Flynn 2007). Within the IgG1 subclass, allelic variant 1*01 was the most stable and already the allele of choice for the majority of clinical mAbs alongside 1*03, 1*07, and 1*08 (Rispens et al. 2014; Richardson et al. 2019). Clinically approved IgG2 mAbs predominantly use 2*01 allele scaffolds (e.g., evolocumab, tezepelumab) although we note crizanlizumab and gevokizumab use the 2*02 allele (Poiron et al. 2010; Wilkinson and Hale 2022). These IgG2 allelic variants possessed unique REES profiles showing evidence of unfolding (increased hydrophobic residue exposure) accompanied by only small changes in structural rigidity, potentially suggesting minimal aggregate formation. Further investigation is required to elucidate the nature of structural changes occurring to produce these REES profiles. Clinical approval for IgG3 antibodies has remained elusive, with the vast genetic diversity between alleles a major concern for stability and potential immunogenicity in therapeutic settings (Salfeld 2007; Jefferis 2007). Our results show a diverse profile of conformational stability between IgG3 allelic variants with 3*11 and 3*17 exhibiting the greatest aggregation propensities and longer hinge region variants demonstrating improved thermal stability (Figure 5f,g). While the first transition temperature for 3*17 was measured to be the highest of the IgG3 variants tested by DSF, we note that the relationship between conformational stability and aggregation propensity is highly complex for multi‐domain antibody structures and transition temperatures do not always align with aggregation rates depending on experimental conditions (Brader et al. 2015). Embracing this naturally occurring sequence variation, particularly within the IgG3 subclass, could aid targeted mAb therapies (Rispens et al. 2014; Richardson et al. 2019; Stapleton et al. 2011) and potentially unlock mAbs that exploit unique functional capabilities (Tay et al. 2016). Despite the presence of allelic mutations distal to the CH1‐VH interface (Figure 2b), further studies using variable domains from clinical antibodies beyond trastuzumab will improve our understanding of variant influence on stability, especially since several reports indicate possible variable domain influence on constant region function (Tang et al. 2021).

Analysis exploiting the conservation of mutations across multiple alleles within the IgG3 subclass provided insights into potential mechanisms behind variant stability rankings. For example, position 435 within IgG3 alleles harbors either a histidine or an arginine and is known to influence binding to the neonatal Fc receptor (Stapleton et al. 2011; DeLano et al. 2000). This position is closely linked to the adjacent position 436 which varies as either tyrosine or phenylalanine. Our observation of greater stability of variants with Arg‐435/Tyr‐436 mutations compared to those possessing Arg‐435/Phe‐436 or His‐435/Tyr‐436 mutations is likely due to a combination of factors. First, Arg‐435 interacts with both Leu‐251 and Ile‐253 (Figure S6), rather than Leu‐251 alone, which could limit the motion of CH2 relative to the CH3 domain and thus create a more rigid structure than variants containing His‐435 (Teplyakov et al. 2013; Shah et al. 2017). Second, we suggest that the hydroxyl group of Tyr‐436 offers the potential for stabilizing hydrogen bonds to form with Ser‐426 and Gln‐438; an interaction not available to Phe‐436. The influence of other mutations on stability was less clear. For instance, IgG3*17 has valine at position 397 located in the CH3 dimerization interface (Figure S6) which has been linked to altered C1q binding, fab‐arm exchange, and propensity to aggregate (Rispens et al. 2014; Natsume et al. 2008). This mutation in combination with Lys‐392 has been previously reported to reduce aggregation in IgG3 structures, albeit in different allelic backgrounds (Saito et al. 2019), in contrast to our observations of higher aggregation propensity. Given these differences, it is likely that single point mutations significantly contribute to overall constant region stability.

To the best of our knowledge, this study represents the first comprehensive interrogation of constant region allelic stability for IgG1, IgG2, and IgG3. We expect further improvements can be made to enhance the resolution of the technique through improvements to spectrophotometers that can perform time‐resolved REES measurements at faster timescales. More broadly, we anticipate REES having utility as a rapid stability‐screening tool for antibody variants containing either natural or engineered diversity.

## 4 MATERIALS AND METHODS

### 4.1 Selection and expression of antibody variants

Exon sequences for 32 human IGHG1, IGHG2, and IGHG3 genes were obtained from the International Immunogenetics Information System® (IMGT) database (Giudicelli et al. 2006). For the purposes of this study, IGHG allele sequences were taken from the IMGT database alone as alleles published on other databases are yet to be robustly verified (Collins et al. 2021). Only allelic sequences with non‐synonymous mutations were selected (Table S1). To meet the requirements of the REES technique (Knight et al. 2020; Kwok et al. 2021), we selected alleles within each subclass containing equal numbers of tryptophan residues. IGHG4 alleles were excluded from analysis due to incomplete sequence information in the CH1 domain or the presence of synonymous mutations.

For each allele, expression constructs were designed, synthesized, and cloned into pTwist CMV BetaGlobulin WPRE Neo vectors (Twist Biosciences, USA). Briefly, constant region allelic sequences were appended to trastuzumab variable heavy sequences with a rabbit IGHG endoplasmic reticulum signal sequence to create full‐length heavy chains. Similarly, a universal light chain was created by joining the trastuzumab variable light sequence to the IGKC*01 kappa constant allele with a rabbit IGKC signal peptide.

Antibodies were expressed by transient transfection using the Expi293 expression system according to the manufacturer's instructions for 25 mL cultures (Thermo Fisher, USA). Expi293 cells were maintained at 37°C, 8% CO_2_ with shaking at 125 rpm for the duration of the experiment. Plasmid DNA was transfected at a 2:1 ratio of light chain to heavy chain, respectively. Expressed antibodies were harvested by collecting supernatants 6 days after transfection by centrifugation at 5000 × *g* for 20 min at 4°C. Supernatants were passed through 0.22 μm filters prior to purification. Gravity flow columns were packed with Protein G sepharose (Thermo Fisher, USA) to a final bed volume of 1 mL. Antibody supernatants were passed through the column three times and washed with 50 mM sodium phosphate buffer, pH 7.5. Antibodies were eluted at pH 2.5 with 4 × 1 mL volumes of formic acid directly into 10 kDa Amicon® Ultra‐15 filter units (Merck Millipore, USA) containing 500 μL of 1 M Ammonium Carbonate pH 8.0. The filter units were centrifuged at 5000 × *g* for 15 min at 4°C and buffer exchanged into 50 mM sodium phosphate pH 7.5. Antibody purity was determined by SDS‐PAGE using 12% cross‐linked acrylamide gels under reducing conditions.

### 4.2 Homology modeling

Homology models of IgG1 allelic variants were built using the Robetta protein structure prediction service (https://robetta.bakerlab.org/) with the comparative modeling setting (Song et al. 2013). The crystal structure of full‐length IgG1 anti‐gp120 mAb (1HZH) was retrieved from PDB and used as the template. The full trastuzumab sequence of each IgG1 constant region allele was threaded onto 1HZH template. Homology models were aligned using PYMOL software and the quality of alignment was determined by calculating the root mean square deviation from 1HZH.

### 4.3 Red edge excitation shift analysis

Antibody stability was assessed using the method established by Knight et al. (Knight et al. 2020) with some minor adaptations. Antibodies were diluted to 150 μg mL^−1^ in 50 mM Tris–HCl buffered saline pH 8.0. Fluorescent measurements were performed using a Hitachi F‐7000 fluorescent spectrometer connected to a circulating water bath set to 10°C. Samples were analyzed in triplicate in a 200 μL micro‐cuvette with a magnetic cuvette stirrer. Dry compressed air was supplied to the chamber to prevent condensation. All samples were equilibrated to 10°C for 5 min prior to measurement. Both excitation and emission slit widths were set to 5 nm. A 3D scan was run with excitation wavelengths from 290 to 310 nm in 1 nm steps and emission wavelengths monitored from 325 to 500 nm in 1 nm steps. A control sample containing only 50 mM Tris–HCl buffered saline pH 8.0 was used for background subtraction and removal of solvent‐induced Raman scattering. Immediately following fluorescent scanning, samples were incubated at 65°C in an Eppendorf thermomixer for 5 h, transferred back to 10°C and allowed to equilibrate for 5 min before repeating the 3D fluorescent scan.

Data processing and analyses were undertaken using R software. Control sample fluorescent scans were averaged to determine the background fluorescence and subtracted from each individual antibody allele scan. The CSM was calculated for each scan using Equation (1):

(1) $$\mathrm{CSM}=\frac{\sum \left(f_{i}\lambda_{\mathrm{Em}}\right)}{\sum \left(f_{i}\right)}$$

where *f*_*i*_ is the measured fluorescence intensity and *λ*_Em_ is the emission wavelength (Knight, 2020). The CSM data was then fit with a thermodynamic model of REES behavior that describes a two‐state transition of tryptophan fluorophores from an excited to a relaxed state (Kwok et al. 2021) (Equation 2):

(2) $$\mathrm{CSM}\left(\lambda_{\mathrm{Ex}}\right)=\;\frac{\mathrm{CSM}\left(\lambda_{\mathrm{Ex}}^{\mathrm{Fc}}\right)+\mathrm{CSM}\left(\lambda_{\mathrm{Ex}}^{R}\right)e^{\left(\frac{∆\mathrm{Gm}\left(\lambda_{\mathrm{Ex}}-\lambda_{\mathrm{Ex}}^{50\%}\right)}{\mathrm{RT}}\right)}}{1+e^{\left(\frac{∆\mathrm{Gm}\left(\lambda_{\mathrm{Ex}}-\lambda_{\mathrm{Ex}}^{50\%}\right)}{\mathrm{RT}}\right)}}$$

where $\mathrm{CSM}\left(\lambda_{\mathrm{Ex}}^{\mathrm{Fc}}\right)$ is the CSM for collective tryptophan emission in an excited state (also known as the Frank‐Codon state), $\mathrm{CSM}\left(\lambda_{\mathrm{Ex}}^{R}\right)$ the CSM for the fully relaxed tryptophan state, $\lambda_{\mathrm{Ex}}$ the excitation wavelength, $\lambda_{\mathrm{Ex}}^{50\%}$ the excitation wavelength at half the maximal CSM between the excited and relaxed states, and ∆*G*_*m*_ is the gradient of the slope describing the change in free energy (∆*G*) of the solvent–fluorophore interaction states sampled during tryptophan relaxation. *R* is the gas constant of 8.3145 J mol^−1^ K^−1^ and *T* is temperature of the fluorescent measurement in K (283.15 K as experiments were conducted at 10°C). The CSM of the relaxed state, $\mathrm{CSM}\left(\lambda_{\mathrm{Ex}}^{R}\right)$, is the maximum wavelength of emission in which the antibodies would be fully solvated and will be the same for all antibodies given the same number of tryptophan residues. This variable was globally fit to all antibody variants and constrained between 387 and 440 nm, a practical range of $\mathrm{CSM}\left(\lambda_{\mathrm{Ex}}^{R}\right)$ values determined by Kwok et al. (2021). Statistical testing for comparative analyses were performed using one‐way ANOVA with Tukey Post‐Hoc multiple comparisons tests and the *p*‐adjusted values were used to determine significance with a *p* = 0.05 cut‐off.

### 4.4 Differential scanning fluorimetry

Differential scanning fluorimetry was used to determine the temperature of unfolding for a subset of antibody alleles (IgG1*01, *11, IgG2*04, *09, IgG3*03, *09, *16, *17). SYPRO orange protein gel stain (Invitrogen) was prepared at 25X concentration with phosphate buffered saline (PBS) pH 7.4. Samples were prepared with 300 μg mL^−1^ antibody, 2.5X final concentration SYPRO orange dye and PBS to a final volume of 25 μL in 0.2 mL optically clear tubes. A buffer‐only control was prepared with SYPRO orange dye and PBS only. All samples were run in triplicate on a Rotor‐Gene 6000 real‐time PCR cycler with 36‐tube ring and Rotor‐Gene Q series software. The temperature was held at 25°C for 1.5 min before beginning the thermal ramp from 25°C to 99°C in 0.2°C increments with a 5 s hold at each step. Excitation was set at 470 nm and emission detected at 555 nm with autogain enabled. Each antibody scan was corrected for background fluorescence by subtracting absorbance from the buffer‐only control. The first derivate was calculated and the maximum value of each first derivative peak was determined to be the thermal melting transition (*T*_m_), equivalent to the inflection point of the DSF curves.

### 4.5 Glycan analysis

To characterize the glycan profiles of each allelic variant, glycans were analyzed by modifying the method reported by Segu et al., (Segu et al. 2020). Antibodies (50 μg) were immobilized on Protein G sepharose resin and treated with PNGase F Rapid (NEB, USA). Enzymatic release was performed at 45°C for 45 min. Released glycans were collected from the supernatant after centrifugation at 4000 × *g* for 2 min and labeled with InstantPC dye (Agilent, USA). The PC dye was prepared following the manufacturer's protocol, added to the glycan sample, and left at room temperature away from light for 10 min. Labeled samples were washed three times with 950 μL acetone and centrifuged at 18,000 × *g* for 5 min. Samples were dried using a centrifugal vacuum evaporator for 20 min at ambient temperature and subsequently stored dry at −20°C before use.

Glycans were resuspended in 25 μL ddH_2_O and transferred to glass vials where 75 μL of acetonitrile was added. Samples were centrifuged at max speed for 1 hr at 4°C to remove any precipitant. HPLC was carried out using Thermo Scientific™ Ultimate™ 3000 UPLC systems with a 1.7 μm 2.1 × 150 mm AQUITY UPLC BEH Amide column (Waters, USA) and fluorescence detection (excitation at 285 nm and emission at 345 nm). The flow rate was 0.5 mL min^−1^. Mobile phase A was 100 mM ammonium formate, pH 4.4 and mobile phase B was 100% acetonitrile. Labeled glycans were separated at 60°C using a gradient elution. After an initial hold of 23% A for up to 25 min to remove the dye front, phase A was increased gradually from 23% to 39% over 42–48 min. The column was cleaned and re‐equilibrated between each run by rapidly increasing A from 39% to 90% over 1 min, holding at 90% A for 5 min, rapidly decreasing A from 90% to 23% over 1 min, and finally holding at 23% A for 6 min. HPLC data was processed using Thermo Fisher Chromeleon software.

LC–MS was performed on a Thermo Scientific Q Exactive Orbitrap mass spectrometer with liquid chromatography conditions as described above. The electrospray ionization voltage was set at 3.5 kV and the capillary maintained at 350°C. The maximum injection time was 100 ms. Sheath gas and auxiliary gas flow rates were held at 25 and 10 mL min^−1^, respectively. Full MS was performed at a resolution of 70,000 and a scan range of 500–2500 *m*/*z* was used to detect glycan species. Mass spectrometry spectral data were processed using Thermo XCalibur FreeStyle software. Glycoforms were assigned to MS peaks manually based on expected mass‐to‐charge ratios of IgG glycoforms (Table S3) (Bereman et al. 2009), taking into account the mass of the conjugated dye (262.3 Da).

## AUTHOR CONTRIBUTIONS

**Annmaree K. Warrender:** Formal analysis (lead); investigation (lead); methodology (equal); visualization (equal); writing – original draft (equal); writing – review and editing (equal). **Jolyn Pan:** Investigation (supporting); writing – review and editing (equal). **Christopher Pudney:** Formal analysis (supporting); methodology (supporting); writing – review and editing (equal). **Vickery Arcus:** Conceptualization (equal); methodology (supporting); writing – review and editing (equal). **William Kelton:** Conceptualization (equal); funding acquisition (lead); investigation (supporting); methodology (equal); project administration (lead); supervision (lead); visualization (equal); writing – original draft (equal); writing – review and editing (equal).

## CONFLICT OF INTEREST STATEMENT

CRP is a director of BLOC Laboratories Limited, which holds a relevant patent (WO2017158371A1).

## Supporting information


**Data S1:** Supporting Information

## Data Availability

The data that supports the findings of this study are available in the supplementary material of this article.
