# Supplementary material for: Constant domain polymorphisms influence monoclonal antibody stability and dynamics
Source: Protein Sci. 2023 Feb 24;32(3):e4589. doi: 10.1002/pro.4589 (PMC9951194; doi:10.1002/pro.4589)
Supplement: Supplementary file 1 — Data S1: Supporting Information [file PRO-32-e4589-s001.pdf]

Constant domain polymorphisms influence monoclonal antibody stability and dynamics

Annmaree Warrender, Joyln Pan, Chris Pudney, Vic Arcus, William Kelton

Supplementary Material

Supplementary Tables

Table S1. Amino acid sequences for antibody variants used in this study

>Herceptin_VL	EMADIQMTQSPSSLSASVGDRVTITCRASQDVNTAVAWYQQKPGKAPKLLIYSASFLY SGVPSRFSGSRSGTDFTLTISSLQPEDFATYYCQQHYTTPPTFGQGTKVEIKR
>IGKC_CL	EMADIQMTQSPSSLSASVGDRVTITCRASQDVNTAVAWYQQKPGKAPKLLIYSASFLY SGVPSRFSGSRSGTDFTLTISSLQPEDFATYYCQQHYTTPPTFGQGTKVEIKRTAAAPSV FIFPPSDEQLKSGTASVVCLLNNFYPREAKVQWKVDNALQSGNSQESVTEQDSKDSTY SLSSTLTLSKADYEKHKVYACEVTHQGLSSPVTKSFNRGEC
>Herceptin_VH	EMAEVQLVESGGGLVQPGGSLRLSCAASGFNIKDTYIHWVRQAPGKGLEWVARIYPT NGYTRYADSVKGRFTISADTSKNTAYLQMNSLRAEDTAVYYCSRWGGDGFYAMDY WGQGTLVTVSS
>IGHG1_01	ASTKGPSVFPLAPSSKSTSGGTAALGCLVKDYFPEPVTVSWNSGALTSGVHTFPAVLQ SSGLYSLSSVVTVPSSSLGTQTYICNVNHKPSNTKVDKKVEPKSCDKTHTCPPCPAPEL LGGPSVFLFPPKPKDTLMISRTPEVTCVVVDVSHEDPEVKFNWYVDGVEVHNAKTKP REEQYNSTYRVVSVLTVLHQDWLNGKEYKCKVSNKALPAPIEKTISKAKGQPREPQV YTLPPSRDELTKNQVSLTCLVKGFYPSDIAVEWESNGQPENNYKTTPPVLDSDGSFFLY SKLTVDKSRWQQGNVFSCSVMHEALHNHYTQKSLSLSPGK
>IGHG1_03	ASTKGPSVFPLAPSSKSTSGGTAALGCLVKDYFPEPVTVSWNSGALTSGVHTFPAVLQ SSGLYSLSSVVTVPSSSLGTQTYICNVNHKPSNTKVDKRVEPKSCDKTHTCPPCPAPEL LGGPSVFLFPPKPKDTLMISRTPEVTCVVVDVSHEDPEVKFNWYVDGVEVHNAKTKP REEQYNSTYRVVSVLTVLHQDWLNGKEYKCKVSNKALPAPIEKTISKAKGQPREPQV YTLPPSREEMTKNQVSLTCLVKGFYPSDIAVEWESNGQPENNYKTTPPVLDSDGSFFL YSKLTVDKSRWQQGNVFSCSVMHEALHNHYTQKSLSLSPGK
>IGHG1_04	ASTKGPSVFPLAPSSKSTSGGTAALGCLVKDYFPEPVTVSWNSGALTSGVHTFPAVLQ SSGLYSLSSVVTVPSSSLGTQTYICNVNHKPSNTKVDKKVEPKSCDKTHTCPPCPAPEL LGGPSVFLFPPKPKDTLMISRTPEVTCVVVDVSHEDPEVKFNWYVDGVEVHNAKTKP REEQYNSTYRVVSVLTVLHQDWLNGKEYKCKVSNKALPAPIEKTISKAKGQPREPQV YTLPPSRDELTKNQVSLTCLVKGFYPSDIAVEWESNGQPENNYKTTPPVLDSDGSFFLY SKLTVDKSRWQQGNIFSCSVMHEALHNHYTQKSLSLSPGK
>IGHG1_07	ASTKGPSVFPLAPSSKSTSGGTAALGCLVKDYFPEPVTVSWNSGALTSGVHTFPAVLQ SSGLYSLSSVVTVPSSSLGTQTYICNVNHKPSNTKVDKKVEPKSCDKTHTCPPCPAPEL LGGPSVFLFPPKPKDTLMISRTPEVTCVVVDVSHEDPEVKFNWYVDGVEVHNAKTKP REEQYNSTYRVVSVLTVLHQDWLNGKEYKCKVSNKALPAPIEKTISKAKGQPREPQV YTLPPSRDELTKNQVSLTCLVKGFYPSDIAVEWESNGQPENNYKTTPPVLDSDGSFFLY SKLTVDKSRWQQGNVFSCSVMHEGLHNHYTQKSLSLSPGK
>IGHG1_08	ASTKGPSVFPLAPSSKSTSGGTAALGCLVKDYFPEPVTVSWNSGALTSGVHTFPAVLQ SSGLYSLSSVVTVPSSSLGTQTYICNVNHKPSNTKVDKRVEPKSCDKTHTCPPCPAPEL LGGPSVFLFPPKPKDTLMISRTPEVTCVVVDVSHEDPEVKFNWYVDGVEVHNAKTKP REEQYNSTYRVVSVLTVLHQDWLNGKEYKCKVSNKALPAPIEKTISKAKGQPREPQV YTLPPSRDELTKNQVSLTCLVKGFYPSDIAVEWESNGQPENNYKTTPPVLDSDGSFFLY SKLTVDKSRWQQGNVFSCSVMHEALHNHYTQKSLSLSPGK

>IGHG1_11	ASTKGPSVFPLAPSSKSTSGGTAALGCLVKDYFPEPVTVSWNSGALTSGVHTFPAVLQ SSGLYSLSSVVTVPSSSLGTQTYICNVNHKPSNTKVDKKVEPKSCDKTHTCPPCPAPEL LGGPSVFLFPPKPKDTLMISRTPEVTCVVVDVSHEDPEVKFNWYVDGVEVHNAKTKP REEQYNSTYRVVSVLTVVHQDWLNGKEYKCKVSNKALPAPIEKTISKAKGQPREPQV YTLPPSRDELTKNQVSLTCLVKGFYPSDIAVEWESNGQPENNYKTTPPVLDSDGSFFLY SKLTVDKSRWQQGNVFSCSVMHEALHNHYTQKSLSLSPGK
>IGHG1_13	ASTKGPSVFPLAPSSKSTSGGTAALGCLVKDYFPEPVTVSWNSGALTSGVHTFPAVLQ SSGLYSLSSVVTVPSSSLGTQTYICNVNHKPSNTKVDKKVEPKSCDKTHTCPPCPAPEL LGGPSVFLFPPKPKDTLMISRTPEVTCVVVDVSHEDPEVKFNWYVDGVEVHNAKTKP REEQFNSTYRVVSVLTVLHQDWLNGKEYKCKVSNKALPAPIEKTISKAKGQPREPQV YTLPPSRDELTKNQVSLTCLVKGFYPSDIAVEWESNGQPENNYKTTPPVLDSDGSFFLY SKLTVDKSRWQQGNVFSCSVMHEALHNHYTQKSLSLSPGK
>IGHG2_01	ASTKGPSVFPLAPCSRSTSESTAALGCLVKDYFPEPVTVSWNSGALTSGVHTFPAVLQS SGLYSLSSVVTVPSSNFGTQTYTCNVDHKPSNTKVDKTVERKCCVECPPCPAPPVAGP SVFLFPPKPKDTLMISRTPEVTCVVVDVSHEDPEVQFNWYVDGVEVHNAKTKPREEQ FNSTFRVVSVLTVVHQDWLNGKEYKCKVSNKGLPAPIEKTISKTKGQPREPQVYTLPP SREEMTKNQVSLTCLVKGFYPSDIAVEWESNGQPENNYKTTPPMLDSDGSFFLYSKLT VDKSRWQQGNVFSCSVMHEALHNHYTQKSLSLSPGK
>IGHG2_02	ASTKGPSVFPLAPCSRSTSESTAALGCLVKDYFPEPVTVSWNSGALTSGVHTFPAVLQS SGLYSLSSVVTVTSSNFGTQTYTCNVDHKPSNTKVDKTVERKCCVECPPCPAPPVAGP SVFLFPPKPKDTLMISRTPEVTCVVVDVSHEDPEVQFNWYVDGMEVHNAKTKPREEQ FNSTFRVVSVLTVVHQDWLNGKEYKCKVSNKGLPAPIEKTISKTKGQPREPQVYTLPP SREEMTKNQVSLTCLVKGFYPSDIAVEWESNGQPENNYKTTPPMLDSDGSFFLYSKLT VDKSRWQQGNVFSCSVMHEALHNHYTQKSLSLSPGK
>IGHG2_04	ASTKGPSVFPLAPCSRSTSESTAALGCLVKDYFPEPVTVSWNSGALTSGVHTFPAVLQS SGLYSLSSVVTVPSSSLGTQTYTCNVDHKPSNTKVDKTVERKCCVECPPCPAPPVAGP SVFLFPPKPKDTLMISRTPEVTCVVVDVSHEDPEVQFNWYVDGVEVHNAKTKPREEQ FNSTFRVVSVLTVVHQDWLNGKEYKCKVSNKGLPAPIEKTISKTKGQPREPQVYTLPP SREEMTKNQVSLTCLVKGFYPSDIAVEWESNGQPENNYKTTPPMLDSDGSFFLYSKLT VDKSRWQQGNVFSCSVMHEALHNHYTQKSLSLSPGK
>IGHG2_06	ASTKGPSVFPLAPCSRSTSESTAALGCLVKDYFPEPVTVSWNSGALTSGVHTFPAVLQS SGLYSLSSVVTVPSSNFGTQTYTCNVDHKPSNTKVDKTVERKCCVECPPCPAPPVAGP SVFLFPPKPKDTLMISRTPEVTCVVVDVSHEDPEVQFNWYVDGVEVHNAKTKPREEQ FNSTFRVVSVLTVVHQDWLNGKEYKCKVSNKGLPAPIEKTISKTKGQPREPQVYTLPP SREEMTKNQVSLTCLVKGFYPSDISVEWESNGQPENNYKTTPPMLDSDGSFFLYSKLT VDKSRWQQGNVFSCSVMHEALHNHYTQKSLSLSPGK
>IGHG2_09	ASTKGPSVFPLAPCSRSTSGGTAALGCLVKDYFPEPVTVSWNSGALTSGVHTFPAVLQ SSGLYSLSSVVTVPSSNFGTQTYTCNVDHKPSNTKVDKTVERKCCVECPPCPAPPVAG PSVFLFPPKPKDTLMISRTPEVTCVVVDVSHEDPEVQFNWYVDGVEVHNAKTKPREE QFNSTFRVVSVLTVVHQDWLNGKEYKCKVSNKGLPAPIEKTISKTKGQPREPQVYTLP PSREEMTKNQVSLTCLVKGFYPSDIAVEWESNGQPENNYKTTPPMLDSDGSFFLYSKL TVDKSRWQQGNVFSCSVMHEALHNHYTQKSLSLSPGK

>IGHG2_11	ASTKGPSVFPLAPCSRSTSESTAALGCLVKDYFPEPVTVSWNSGALTSGVHTFPAVLQS SGLYSLSSVVTVPSSNFGTQTYTCNVDHKPSNTKVDKTVERKCCVECPPCPAPPVAGP SVFLFPPKPKDTLMISRTPEVTCVVVDVSHEDPEVQFNWYVDGVEVHNAKTKPREEQ FNSTFRVVSVLTVLHQDWLNGKEYKCKVSNKGLPAPIEKTISKTKGQPREPQVYTLPP SREEMTKNQVSLTCLVKGFYPSDIAVEWESNGQPENNYKTTPPMLDSDGSFFLYSKLT VDKSRWQQGNVFSCSVMHEALHNHYTQKSLSLSPGK
>IGHG2_15	ASTKGPSVFPLAPCSRSTSESTAALGCLVKDYFPEPVTVSWNSGALTSGVHTFPAVLQS SGLYSLSSVVTVPSSNFGTQTYTCNVDHKPSNTKVDKTVERKCCVECPPCPAPPVAGP SVFLFPPKPKDTLMISRTPEVTCVVVDVSHEDPEVQFNWYVDGVEVHNAKTKPREEQ FNSTFRVVSVLTVVHQDWLNGKEYKCKVSNKGLPAPIEKTISKTKGQPREPQVYTLPP SREEMTKNQVSLTCLVKGFYPSDIAVEWESNGQPENNYNTTPPMLDSDGSFFLYSKLT VDKSRWQQGNVFSCSVMHEALHNHYTQKSLSLSPGK
>IGHG3_01	ASTKGPSVFPLAPCSRSTSGGTAALGCLVKDYFPEPVTVSWNSGALTSGVHTFPAVLQ SSGLYSLSSVVTVPSSSLGTQTYTCNVNHKPSNTKVDKRVELKTPLGDTTHTCPRCPEP KSCDTPPPCPRCPEPKSCDTPPPCPRCPEPKSCDTPPPCPRCPAPELLGGPSVFLFPPKPK DTLMISRTPEVTCVVVDVSHEDPEVQFKWYVDGVEVHNAKTKPREEQYNSTFRVVSV LTVLHQDWLNGKEYKCKVSNKALPAPIEKTISKTKGQPREPQVYTLPPSREEMTKNQV SLTCLVKGFYPSDIAVEWESSGQPENNYNTTPPMLDSDGSFFLYSKLTVDKSRWQQGN IFSCSVMHEALHNRFTQKSLSLSPGK
>IGHG3_03	ASTKGPSVFPLAPCSRSTSGGTAALGCLVKDYFPEPVTVSWNSGALTSGVHTFPAVLQ SSGLYSLSSVVTVPSSSLGTQTYTCNVNHKPSNTKVDKRVELKTPLGDTTHTCPRCPEP KSCDTPPPCPRCPEPKSCDTPPPCPRCPAPELLGGPSVFLFPPKPKDTLMISRTPEVTCVV VDVSHEDPEVQFKWYVDGVEVHNAKTKPREEQYNSTFRVVSVLTVLHQDWLNGKE YKCKVSNKALPAPIEKTISKTKGQPREPQVYTLPPSREEMTKNQVSLTCLVKGFYPSDI AVEWESSGQPENNYNTTPPVLDSDGSFFLYSRLTVDKSRWQEGNVFSCSVMHEALHN RFTQKSLSLSPGK
>IGHG3_04	ASTKGPSVFPLAPCSRSTSGGTAALGCLVKDYFPEPVTVSWNSGALTSGVHTFPAVLQ SSGLYSLSSVVTVPSSSLGTQTYTCNVNHKPSNTKVDKRVELKTPLGDTTHTCPRCPEP KSCDTPPPCPRCPAPELLGGPSVFLFPPKPKDTLMISRTPEVTCVVVDVSHEDPEVQFK WYVDGVEVHNAKTKPREEQYNSTFRVVSVLTVLHQDWLNGKEYKCKVSNKALPAPI EKTISKTKGQPREPQVYTLPPSREEMTKNQVSLTCLVKGFYPSDIAVEWESSGQPENNY NTTPPMLDSDGSFFLYSKLTVDKSRWQQGNIFSCSVMHEALHNRFTQKSLSLSPGK
>IGHG3_06	ASTKGPSVFPLAPCSRSTSGGTAALGCLVKDYFPEPVTVSWNSGALTSGVHTFPAVLQ SSGLYSLSSVVTVPSSSLGTQTYTCNVNHKPSNTKVDKRVELKTPLGDTTHTCPRCPEP KSCDTPPPCPRCPEPKSCDTPPPCPRCPEPKSCDTPPPCPRCPAPELLGGPSVFLFPPKPK DTLMISRTPEVTCVVVDVSHEDPEVQFKWYVDGVEVHNAKTKPREEQYNSTFRVVSV LTVLHQDWLNGKEYKCKVSNKALPAPIEKTISKTKGQPREPQVYTLPPSREEMTKNQV SLTCLVKGFYPSDIAVEWESSGQPENNYKTTPPMLDSDGSFFLYSKLTVDKSRWQQGN IFSCSVMHEALHNRFTQKSLSLSPGK
>IGHG3_08	ASTKGPSVFPLAPCSRSTSGGTAALGCLVKDYFPEPVTVSWNSGALTSGVHTFPAVLQ SSGLYSLSSVVTVPSSSLGTQTYTCNVNHKPSNTKVDKRVELKTPLGDTTHTCPRCPEP KSCDTPPPCPRCPEPKSCDTPPPCPRCPEPKSCDTPPPCPRCPAPELLGGPSVFLFPPKPK DTLMISRTPEVTCVVVDVSHEDPEVQFKWYVDGVEVHNAKTKPREEQYNSTFRVVSV

	LTVLHQDWLNGKEYKCKVSNKALPAPIEKTISKTKGQPREPQVYTLPPSREEMTKNQV SLTCLVKGFYPSDIAVEWESNGQPENNYNTTPPMLDSDGSFFLYSKLTVDKSRWQQG NIFSCSVMHEALHNRFTQKSLSLSPGK
>IGHG3_09	ASTKGPSVFPLAPCSRSTSGGTAALGCLVKDYFPEPVTVSWNSGALTSGVHTFPAVLQ SSGLYSLSSVVTVPSSSLGTQTYTCNVNHKPSNTKVDKRVELKTPLGDTTHTCPRCPEP KSCDTPPPCPRCPEPKSCDTPPPCPRCPEPKSCDTPPPCPRCPAPELLGGPSVFLFPPKPK DTLMISRTPEVTCVVVDVSHEDPEVQFKWYVDGVEVHNAKTKPREEQYNSTFRVVSV LTVVHQDWLNGKEYKCKVSNKALPAPIEKTISKTKGQPREPQVYTLPPSREEMTKNQ VSLTCLVKGFYPSDIAVEWESSGQPENNYNTTPPMLDSDGSFFLYSKLTVDKSRWQQG NIFSCSVMHEALHNRFTQKSLSLSPGK
>IGHG3_11	ASTKGPSVFPLAPCSRSTSGGTAALGCLVKDYFPEPVTVSWNSGALTSGVHTFPAVLQ SSGLYSLSSVVTVPSSSLGTQTYTCNVNHKPSNTKVDKRVELKTPLGDTTHTCPRCPEP KSCDTPPPCPRCPEPKSCDTPPPCPRCPEPKSCDTPPPCPRCPAPELLGGPSVFLFPPKPK DTLMISRTPEVTCVVVDVSHEDPEVQFKWYVDGVEVHNAKTKPREEQFNSTFRVVSV LTVLHQDWLNGKEYKCKVSNKALPAPIEKTISKTKGQPREPQVYTLPPSREEMTKNQV SLTCLVKGFYPSDIAVEWESSGQPENNYNTTPPMLDSDGSFFLYSKLTVDKSRWQQGN IFSCSVMHEALHNRFTQKSLSLSPGK
>IGHG3_12	ASTKGPSVFPLAPCSRSTSGGTAALGCLVKDYFPEPVTVSWNSGALTSGVHTFPAVLQ SSGLYSLSSVVTVPSSSLGTQTYTCNVNHKPSNTKVDKRVELKTPLGDTTHTCPRCPEP KSCDTPPPCPRCPEPKSCDTPPPCPRCPAPELLGGPSVFLFPPKPKDTLMISRTPEVTCVV VDVSHEDPEVQFKWYVDGVEVHNAKTKPREEQFNSTFRVVSVLTVLHQDWLNGKEY KCKVSNKALPAPIEKTISKTKGQPREPQVYTLPPSREEMTKNQVSLTCLVKGFYPSDIA VEWESSGQPENNYNTTPPMLDSDGSFFLYSKLTVDKSRWQQGNIFSCSVMHEALHNR FTQKSLSLSPGK
>IGHG3_13	ASTKGPSVFPLAPCSRSTSGGTAALGCLVKDYFPEPVTVSWNSGALTSGVHTFPAVLQ SSGLYSLSSVVTVPSSSLGTQTYTCNVNHKPSNTKVDKRVELKTPLGDTTHTCPRCPEP KSCDTPPPCPRCPEPKSCDTPPPCPRCPEPKSCDTPPPCPRCPAPELLGGPSVFLFPPKPK DTLMISRTPEVTCVVVDVSHEDPEVQFKWYVDGVEVHNAKTKPREEQYNSTFRVVSV LTVLHQDWLNGKEYKCKVSNKALPAPIEKTISKTKGQPREPQVYTLPPSREEMTKNQV SLTCLVKGFYPSDIAVEWESSGQPENNYKTTPPMLDSDGSFFLYSKLTVDKSRWQEGN IFSCSVMHEALHNRFTQKSLSLSPGK
>IGHG3_14	ASTKGPSVFPLAPCSRSTSGGTAALGCLVKDYFPEPVTVSWNSGALTSGVHTFPAVLQ SSGLYSLSSVVTVPSSSLGTQTYTCNVNHKPSNTKVDKRVELKTPLGDTTHTCPRCPEP KSCDTPPPCPRCPEPKSCDTPPPCPRCPEPKSCDTPPPCPRCPAPELLGGPSVFLFPPKPK DTLMISRTPEVTCVVVDVSHEDPEVQFKWYVDGVEVHNAKTKLREEQYNSTFRVVSV LTVLHQDWLNGKEYKCKVSNKALPAPIEKTISKTKGQPREPQVYTLPPSREEMTKNQV SLTCLVKGFYPSDIAVEWESNGQPENNYNTTPPMLDSDGSFFLYSKLTVDKSRWQQG NIFSCSVMHEALHNRYTQKSLSLSPGK
>IGHG3_15	ASTKGPSVFPLAPCSRSTSGGTAALGCLVKDYFPEPVTVSWNSGALTSGVHTFPAVLQ SSGLYSLSSVVTVPSSSLGTQTYTCNVNHKPSNTKVDKRVELKTPLGDTTHTCPRCPEP KSCDTPPPCPRCPEPKSCDTPPPCPRCPEPKSCDTPPPCPRCPAPELLGGPSVFLFPPKPK DTLMISRTPEVTCVVVDVSHEDPEVQFKWYVDGVEVHNAKTKLREEQYNSTFRVVSV LTVLHQDWLNGKEYKCKVSNKALPAPIEKTISKTKGQPREPQVYTLPPSREEMTKNQV

	SLTCLVKGFYPSDIAVEWESNGQPENNYKTTPPMLDSDGSFFLYSKLTVDKSRWQQG NIFSCSVMHEALHNRYTQKSLSLSPGK
>IGHG3_16	ASTKGPSVFPLAPCSRSTSGGTAALGCLVKDYFPEPVTVSWNSGALTSGVHTFPAVLQ SSGLYSLSSVVTVPSSSLGTQTYTCNVNHKPSNTKVDKRVELKTPLGDTTHTCPRCPEP KSCDTPPPCPRCPEPKSCDTPPPCPRCPEPKSCDTPPPCPRCPAPELLGGPSVFLFPPKPK DTLMISRTPEVTCVVVDVSHEDPEVQFKWYVDGVEVHNAKTKLREEQYNSTFRVVSV LTVLHQDWLNGKEYKCKVSNKALPAPIEKTISKAKGQPREPQVYTLPPSREEMTKNQ VSLTCLVKGFYPSDIAVEWESNGQPENNYNTTPPMLDSDGSFFLYSKLTVDKSRWQQ GNIFSCSVMHEALHNRYTQKSLSLSPGK
>IGHG3_17	ASTKGPSVFPLAPCSRSTSGGTAALGCLVKDYFPEPVTVSWNSGALTSGVHTFPAVLQ SSGLYSLSSVVTVPSSNFGTQTYTCNVNHKPSNTKVDKRVELKTPLGDTTHTCPRCPEP KSCDTPPPCPRCPEPKSCDTPPPCPRCPAPELLGGPSVFLFPPKPKDTLMISRTPEVTCVV VDVSHEDPEVQFKWYVDGVEVHNAKTKPREEQYNSTFRVVSVLTVLHQDWLNGKE YKCKVSNKALPAPIEKTISKTKGQPREPQVYTLPPSREEMTKNQVSLTCLVKGFYPSDI AMEWESSGQPENNYKTTPPVLDSDGSFFLYSKLTVDKSRWQQGNIFSCSVMHEALHN HYTQKSLSLSPGK
>IGHG3_20	ASTKGPSVFPLAPCSRSTSGGTAALGCLVKDYFPEPVTVSWNSGALTSGVHTFPAVLQ SSGLYSLSSVVTVPSSSLGTQTYTCNVNHKPSNTKVDKRVELKTPLGDTTHTCPRCPEP KSCDTPPPCPRCPEPKSCDTPPPCPRCPEPKSCDTPPPCPRCPAPELLGGPSVFLFPPKPK DTLMISRTPEVTCVVVDVSHEDPEVQFKWYVDGVEVHNAKTKLREEQYNSTFRVVSV LTVLHQDWLNGKEYKCKVSNKALPAPIEKTISKTKGQPREPQVYTLPPSREEMTKNQV SLTCLVKGFYPSDIAVEWESNGQRENNYNTTPPMLDSDGSFFLYSKLTVDKSRWQQG NIFSCSVMHEALHNRYTQKSLSLSPGK
>IGHG3_22	ASTKGPSVFPLAPCSRSTSGGTAALGCLVKDYFPEPVTVSWNSGALTSGVHTFPAVLQ SSGLYSLSSVVTVPSSSLGTQTYTCNVNHKPSNTKVDKRVELKTPLGDTTHTCPRCPEP KSCDTPPPCPRCPEPKSCDTPPPCPRCPEPKSCDTPPPCPRCPAPELLGGPSVFLFPPKPK DTLMISRTPEVTCVVVDVSHEDPEVQFKWYVDGVEVHNAKTKLREEQYNSTFRVVSV LTVLHQDWLNGKEYKCKVSNKALPAPIEKTISKTKGQPREPQVYTLPPSREEMTKNQV SLTCLVKGFYPSDIAVEWESNGQPENNYNTTPPMLDSDGSFFLYSKLTVDKSRWQQG NIFSCSVMHEALHNHYTQKSLSLSPGK
>IGHG3_24	ASTKGPSVFPLAPCSRSTSGGTAALGCLVKDYFPEPVTVSWNSGALTSGVHTFPAVLQ SSGLYSLSSVVTVPSSSLGTQTYTCNVNHKPSNTKVDKRVELKTPLGDTTHTCPRCPEP KSCDTPPPCPRCPEPKSCDTPPPCPRCPEPKSCDTPPPCPRCPAPELLGGPSVFLFPPKPK DTLMISRTPEVTCVVVDVSHEDPEVQFKWYVDGVEVHNAKTKPREEQYNSTFRVVSV LTVLHQDWLNGKEYKCKVSNKALPAPIEKTISKTKGQPREPQVYTLPPSREEMTKNQV SLTCLVKGFYPSDIAVEWESNGQPENNYKTTPPMLDSDGSFFLYSKLTVDKSRWQQG NIFSCSVMHEALHNRYTQKSLSLSPGK
>IGHG3_25	ASTKGPSVFPLAPCSRSTSGGTAALGCLVKDYFPEPVTVSWNSGALTSGVHTFPAVLQ SSGLYSLSSVVTVPSSSLGTQTYTCNVNHKPSNTKVDKRVELKTPLGDTTHTCPRCPEP KSCDTPPPCPRCPEPKSCDTPPPCPRCPEPKSCDTPPPCPRCPAPELLGGPSVFLFPPKPK DTLMISRTPEVTCVVVDVSHEDPEVKFKWYVDGVEVHNAKTKLREEQYNSTFRVVSV LTVLHQDWLNGKEYKCKVSNKALPAPIEKTISKTKGQPREPQVYTLPPSREEMTKNQV

	SLTCLVKGFYPSDIAVEWESNGQPENNYNTTPPMLDSDGSFFLYSKLTVDKSRWQQG<br>NIFSCSVMHEALHNRYTQKSLSLSPGK
>IGHG3_26	ASTKGPSVFPLAPCSRSTSGGTAALGCLVKDYFPEPVTVSWNSGALTSGVHTFPAVLQ<br>SSGLYSLSSVVTVPSSSLGTQTYTCNVNHKPSNTKVDKRVELKTPLGDTTHTCPRCPEP<br>KSCDTPPPCPRCPEPKSCDTPPPCPRCPEPKSCDTPPPCPRCPAPELLGGPSVFLFPPKPK<br>DTLMISRTPEVTCVVVDVSHEDPEVQFKWYVDGVEVHNAKTKPREEQFNSTFRVVSV<br>LTVLHQDWLNGKEYKCKVSNKGLPAPIEKTISKTKGQPREPQVYTLPPSREEMTKNQV<br>SLTCLVKGFYPSDIAVEWESSGQPENNYNTTPPMLDSDGSFFLYSKLTVDKSRWQQGN<br>IFSCSVMHEALHNRFTQKSLSLSPGK

Table S2. RMSD scores for the alignment of IgG1 structural homology models to 1HZH in PYMOL

Allele	RMSD score	Atoms aligned
1*01	0.707	1195
1*03	1.634	1271
1*04	1.248	1264
1*07	1.054	1264
1*08	0.600	1082
1*11	0.865	1237
1*13	1.856	1288

Table S3. Relative glycan species abundance as a percentage for each antibody variant determined by HPLC-MS. Blank cells indicate the glycan species was not detected.

		IgG1						
**Glycoforms**	**Theoretical mass [M+2H][1]**	***01**	***03**	***04**	***07**	***08**	***11**	***13**
G0-2GlcNAc	911.335						4.47	0.09
G0-GlcNAc	1114.415	0.30	0.53	0.30	0.15	0.23	1.12	0.76
G0 F-GlcNAc	1260.473	4.69	3.34	3.59	3.45	4.93	7.01	3.88
G0	1317.494		0.10		0.11		0.35	0.35
G1 + GlcNac	1682.626							
G1 F-GlcNAc a	1421.525855	0.43	0.41	0.45		0.21	0.63	
G1-GlcNAc	1276.467	0.00	0.31	0.45	0.19			0.24
G0 F	1463.552	46.40	35.55	35.56	31.59	29.71	36.15	44.73
M5 F	1380.499085	0.00	0.28	0.38	0.25	0.22	0.33	0.08
M5	1235.441	8.18	8.97	10.72	7.87	12.42	15.88	7.94
G1 F-GlcNAc b	1421.525855	1.13	1.35	1.39	1.60	0.87	0.53	1.97
G1	1479.547				0.38			0.07
G0 F+ GlcNac	1666.631							0.09
G1 Fa	1625.605	16.67	20.63	19.55	20.84	16.47	13.13	10.40
G1 Fb	1625.605	6.53	7.15	6.60	7.18	4.80	3.96	10.75
G1 F+ GlcNAc a	1828.6839						2.18	
M6	1397.494	0.80	2.68	3.38	2.46	2.89	0.72	1.43
G0 F- GlcNac+SA	1713.6205			0.20				0.20
G1-GlcNac+SA	1566.563185	0.21	0.24	0.22			2.48	
G1 F+ GlcNAc b	1828.6839				0.49	0.66	0.52	0.17
G2 F	1787.6573	3.36	5.68	5.18	6.31	4.49	1.98	2.57
G1 +SA+M1	1729.6154							
M7	1559.546785	0.31	1.41	1.66	1.18	2.78	0.25	1.07
G1 F+ SA	1916.6999							
G2 + SA	1932.6848						1.08	
M8	1721.599585					2.14		0.56
G2 F+ SA	2078.7527				0.16		0.13	0.07
G1 F+ SA+M2	2037.726685							0.16

[1]Bereman, M. S., Young, D. D., Deiters, A., & Muddiman, D. C. (2009). Development of a robust and high throughput method for profiling N-linked Glycans derived from plasma glycoproteins by NanoLC− FTICR mass spectrometry. *Journal of proteome research*, *8*(7), 3764-3770.

	IgG2						
**Glycoforms**	***01**	***02**	***04**	***06**	***09**	***11**	***15**
G0-2GlcNAc					3.59		
G0-GlcNAc		0.75	0.53	0.43	0.67		0.88
G0 F-GlcNAc	4.34	7.04	4.55	5.17	6.54	3.93	4.57
G0				0.31			
G1 + GlcNac							
G1 F-GlcNAc a	1.42	0.39	0.60	0.46			
G1-GlcNAc	0.44				0.41	0.15	0.13
G0 F	32.57	36.10	31.84	29.15	34.44	28.93	35.75
M5 F	0.05	0.37					
M5	13.66	17.72	16.19	12.62	14.66	15.62	12.25
G1 F-GlcNAc b	1.91	0.91	1.58	2.26	2.72	1.93	1.10
G1							
G0 F+ GlcNac							
G1 Fa	6.46	6.15	7.06	6.11	5.94	5.61	6.97
G1 Fb	7.51	6.59	7.39	5.83	7.37	6.78	7.02
G1 F+ GlcNAc a	0.05						
M6	3.26	0.59	0.52	0.94	1.04	0.11	4.08
G0 F- GlcNac+SA							
G1-GlcNac+SA							
G1 F+ GlcNAc b	0.23						
G2 F	1.86	2.03	2.57	2.17	1.18	1.08	2.22
G1+ SA+M1							
M7	2.07	1.91	3.40	1.32	1.59	0.39	1.96
G1 F+ SA							
G2 + SA							
M8	1.99	1.35	3.07	1.04	1.60	3.07	1.17
G2 F +SA							
G1F+SA+M2							

	IgG3										
**Glycoforms**	***01**	***03**	***04**	***06**	***08**	***09**	***11**	***12**	***13**	***14**	***15**
G0-2GlcNAc	0.29										
G0-GlcNAc	0.55	0.59	0.44	0.60	0.74	0.18		0.13		0.29	0.28
G0 F-GlcNAc	2.22	2.49	2.19	3.41	3.88	2.03	1.35	2.68	1.52	4.89	2.23
G0	1.62	0.42	0.08	0.18			0.21	0.10	0.23	1.64	0.17
G1 + GlcNac	0.39	0.28									
G1 F-GlcNAc a					0.25	0.12			0.25		
G1-GlcNAc	0.26	0.18					0.71	0.59			
G0 F	40.55	39.47	46.60	40.42	40.64	33.32	42.84	57.57	45.15	40.48	44.37
M5 F											
M5	9.76	9.17	7.88	8.41	10.86	9.30	9.21	6.69	6.07	6.91	5.69
G1 F-GlcNAc b	0.43	0.09	0.29	1.16	0.09	0.32	0.69	0.36	0.33		0.37
G1		0.29	0.18	0.14						0.29	
G0 F+ GlcNac											
G1 Fa	15.32	17.97	17.20	16.45	15.61	15.88	9.57	9.27	13.38	17.22	17.52
G1 Fb	6.07	7.66	7.72	8.48	6.08	6.48	10.26	11.09	7.26	8.81	10.51
G1 F+ GlcNAc a		0.43									
M6	1.78	3.85	1.83	1.53	2.31	2.89	4.20	1.17	0.75	1.37	0.36
G0 F- GlcNac+SA											
G1-GlcNac+SA		0.13									
G1 F+ GlcNAc b		0.36					0.18	0.14	0.28	0.59	0.77
G2 F	3.60	5.09	3.86	5.08	3.47	5.08	3.55	2.82	3.68	5.29	6.17
G1+ SA+M1		0.36									
M7		1.43									0.42
G1 F+ SA								0.73			
G2 + SA		0.38						0.25			
M8	0.70	0.82	0.25	1.22	0.91	0.64	2.26		0.75	0.32	0.40
G2 F +SA								0.18			
G1F+SA+M2											

	IgG3 (continued)									
**Glycoforms**	***16**	***17**	***18**	***19**	***20**	***22**	***23**	***24**	***25**	***26**
G0-2GlcNAc										
G0-GlcNAc		0.38	0.36	1.76	0.37		0.22	0.59	0.10	2.52
G0 F-GlcNAc	1.48	3.41	1.71	1.63	1.81	1.74	2.01	3.36	1.68	1.90
G0	0.11	0.17	0.27		0.26	0.31	0.09	0.32	0.16	
G1 + GlcNac										
G1 F-GlcNAc a	0.33									
G1-GlcNAc		0.26	1.12							
G0 F	41.93	48.37	40.56	38.02	39.26	40.43	42.84	44.53	37.46	49.66
M5 F										
M5	7.89	7.24	1.76	2.24	8.53	6.97	4.59	7.84	8.32	9.36
G1 F-GlcNAc b		0.60	0.23	0.14	0.27			0.43		0.19
G1	0.16									
G0 F+ GlcNac			0.30		0.37	0.23	0.35	0.25	0.35	0.34
G1 Fa	19.20	16.59	22.32	25.01	18.57	19.71	23.01	15.28	16.67	8.57
G1 Fb	8.27	7.71	8.79	9.28	9.09	8.69	9.10	7.99	8.54	10.20
G1 F+ GlcNAc a						0.15	0.11		0.07	
M6	2.57	0.51	0.09	0.13	1.92	2.88	0.38	0.83	1.46	1.99
G0 F- GlcNac+SA										
G1-GlcNac+SA			1.18							
G1 F+ GlcNAc b	0.74	0.35		1.41		0.69	1.40	0.45	0.89	0.26
G2 F	6.45	3.95	7.92	10.43	6.97	6.24	8.22	4.26	6.50	2.94
G1+ SA+M1										
M7	0.97					1.09			0.70	1.19
G1 F+ SA					1.15					
G2 + SA										
M8	0.35	0.21	0.52	0.60	0.44	0.64	0.49	0.84		0.49
G2 F +SA	0.54								0.40	
G1F+SA+M2										

Table S4. Correlation values between glycoforms and change in CSMFc and ΔG

		IgG1		IgG2		IgG3	
**Glycoform**	**Measurement**	**R**	**p-value**	**R**	**p-value**	**R**	**p-value**
G0 F	$\Delta CSM(\lambda_{Ex}^{Fc})$	-0.66	0.10	0.77	0.04	0.07	0.77
	$\Delta\Delta G_m$	-0.14	0.76	0.02	0.97	0.22	0.38
G1 Fa	$\Delta CSM(\lambda_{Ex}^{Fc})$	0.12	0.80	0.59	0.17	-0.32	0.19
	$\Delta\Delta G_m$	0.29	0.53	-0.47	0.29	-0.35	0.16
G1 Fb	$\Delta CSM(\lambda_{Ex}^{Fc})$	0.02	0.97	-0.06	0.90	-0.25	0.32
	$\Delta\Delta G_m$	-0.30	0.51	0.04	0.93	0.13	0.60
M5	$\Delta CSM(\lambda_{Ex}^{Fc})$	0.26	0.57	-0.04	0.94	0.25	0.31
	$\Delta\Delta G_m$	0.04	0.93	-0.18	0.70	0.15	0.55

Supplementary Figures

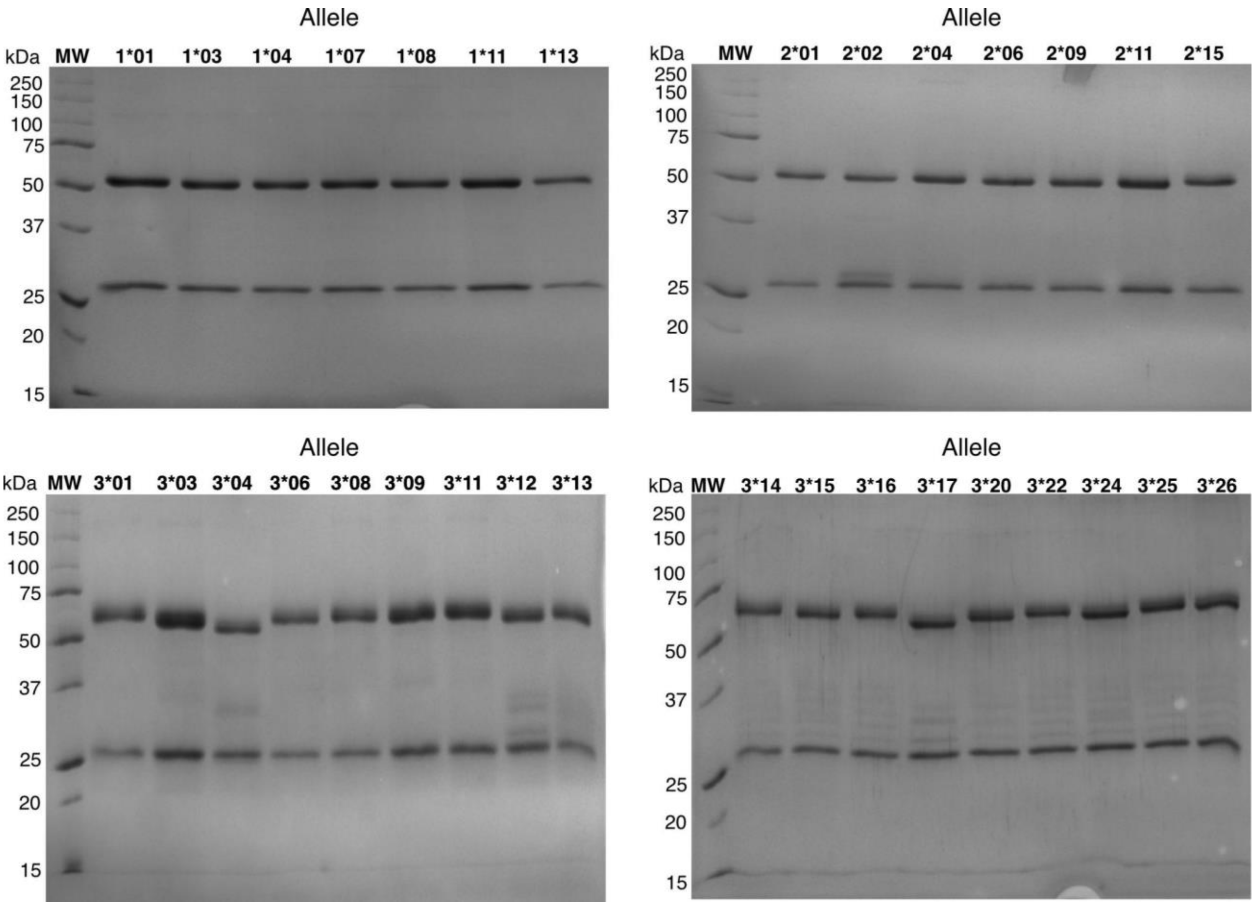

Figure S1. SDS-PAGE gels showing >95% purity of antibody samples following expression and purification. The gels were prepared with 12% cross-linked acrylamide and run under reducing conditions. Expected band size for antibody heavy chain is 50-65 kDa depending on the variant and 25 kDa for the light chain.

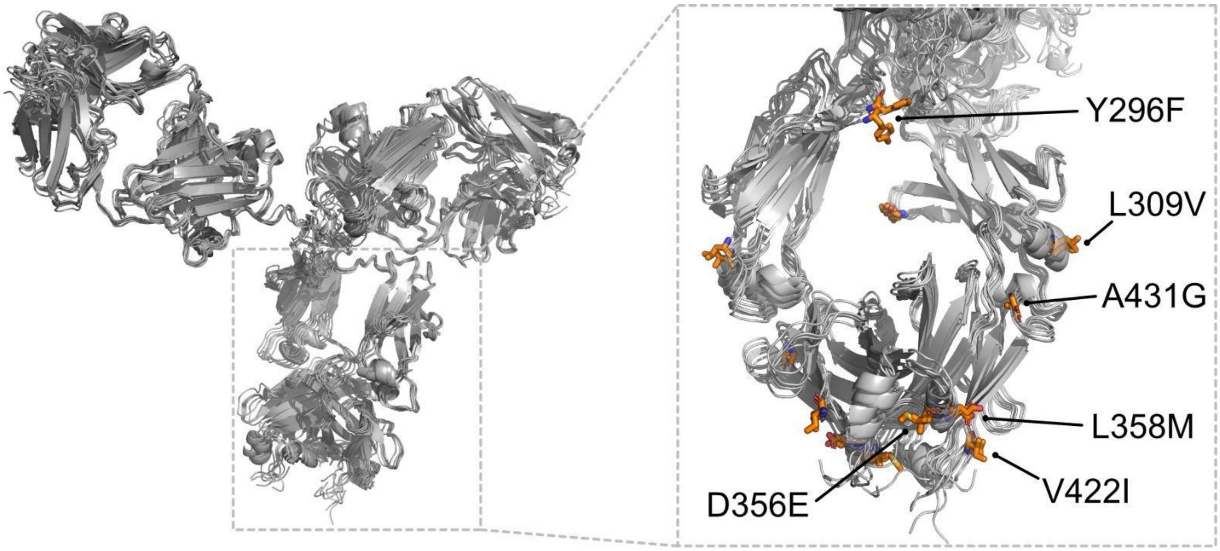

Figure S2. Structural homology models of IgG1 produced using Robetta software. Amino acid sequences were threaded onto PDB structure 1HZH and alignment was analyzed in PYMOL. Aside from differences in the rotation of side chains, there are few differences in the structure of the antibody backbone.

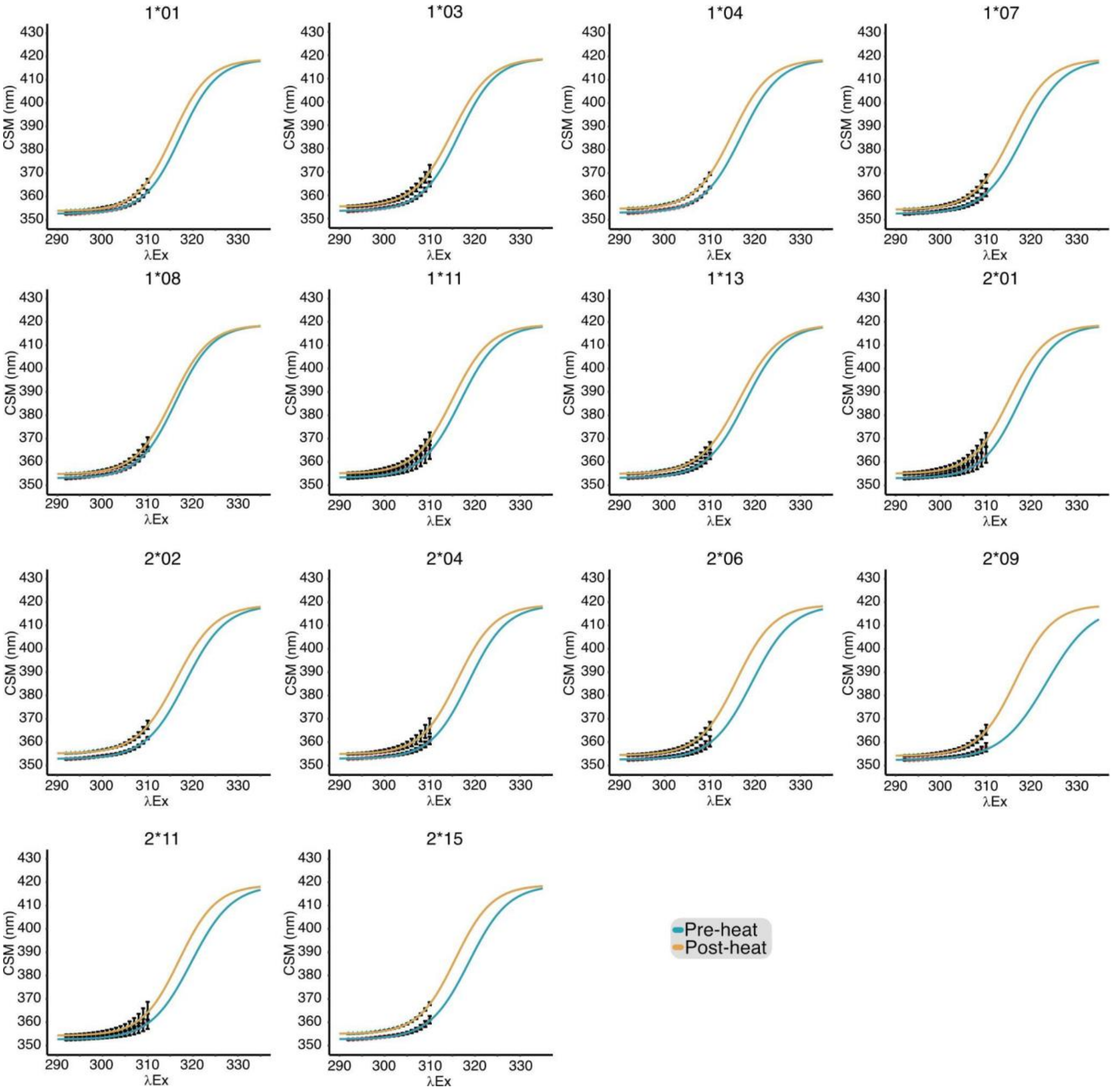

Figure S3. CSM data for each antibody variant before (pre-heat) and after (post-heat) heating. Data was collected in triplicate. The average CSM values are plotted with error bars showing the standard deviation. The solid lines are the fits of Equation 2 to the CSM data.

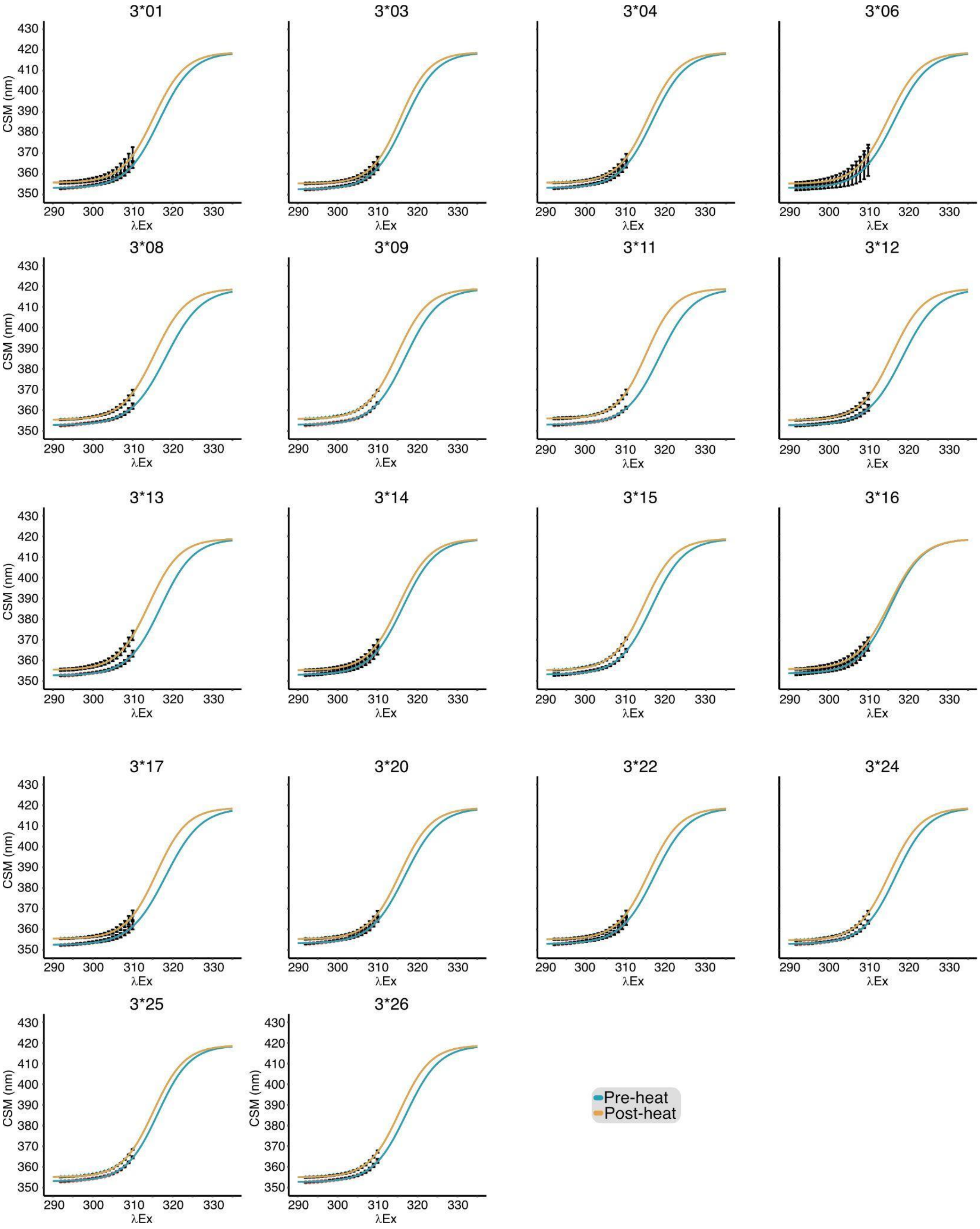

Figure S3 (continued). CSM data for each antibody variant before (pre-heat) and after (post-heat) heating. Data was collected in triplicate. The average CSM values are plotted with error bars showing the standard deviation. The solid lines are the fits of Equation 2 to the CSM data.

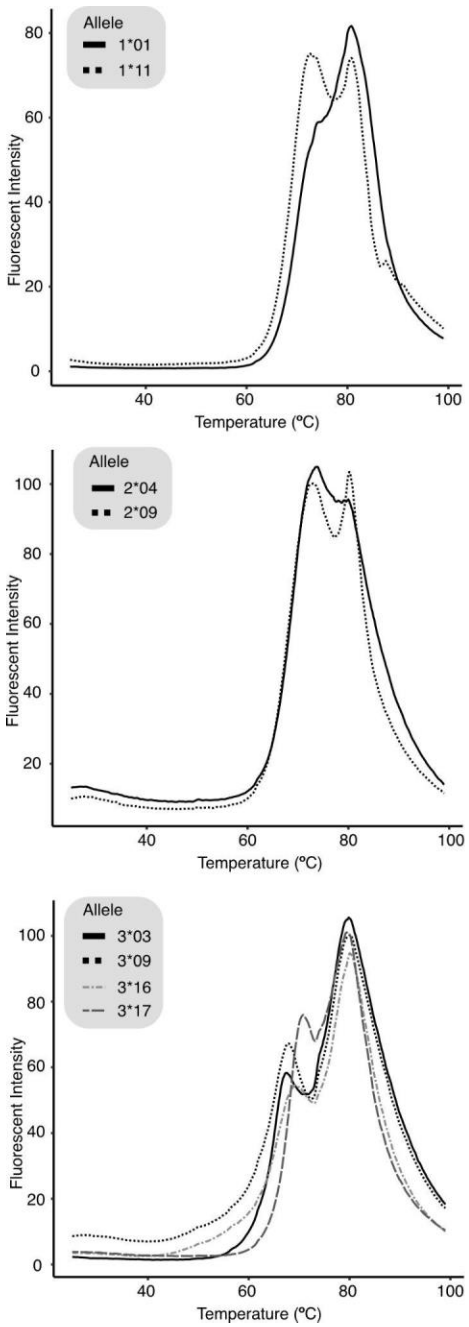

Figure S4. Thermal unfolding (melt) curves measured by differential scanning fluorimetry using SYPRO orange dye.

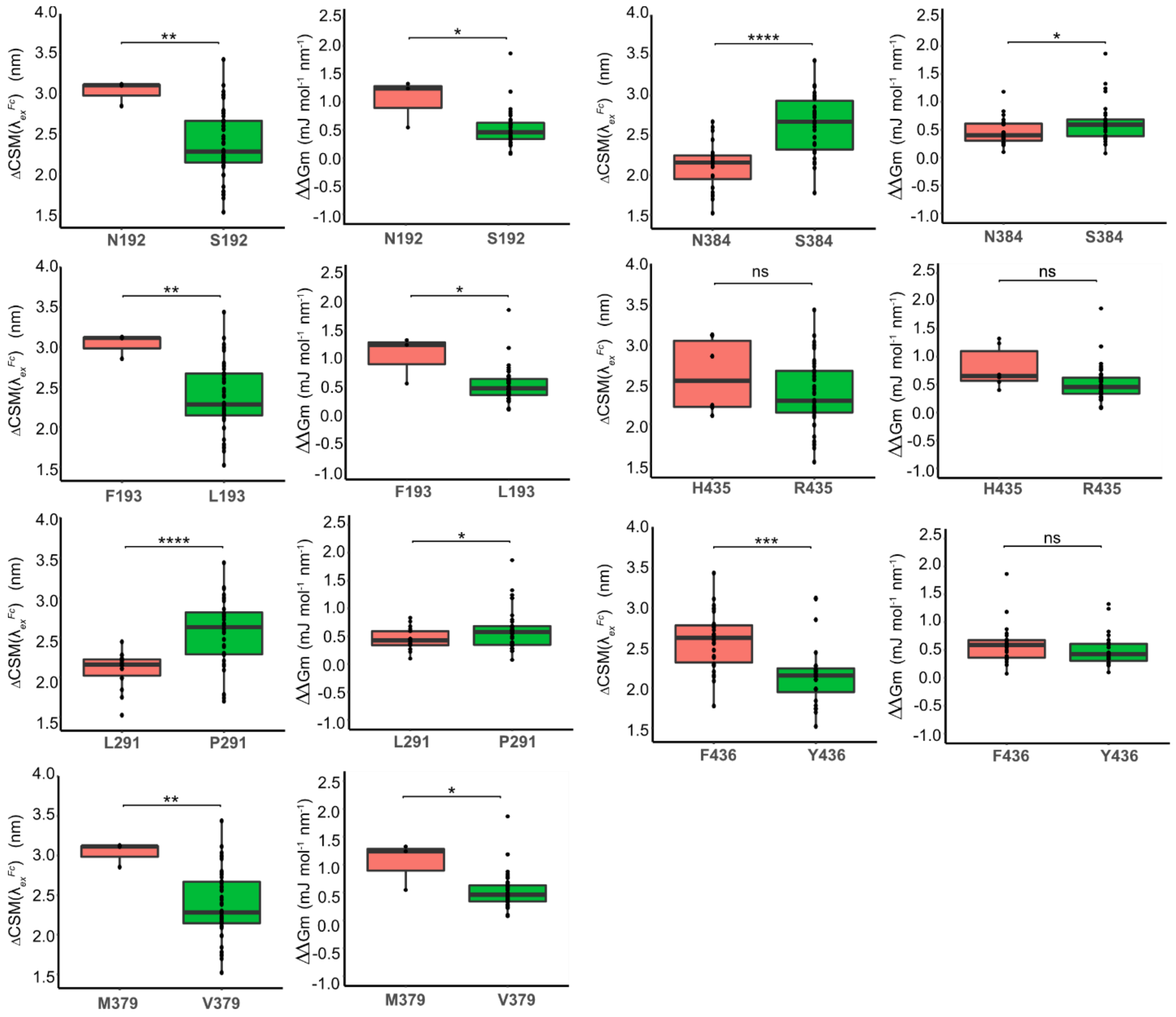

Figure S5. Allelic mutations show trends in REES stability measurements. Boxplots showing the magnitude of change in $\boldsymbol{CSM(\lambda_{Ex}^{Fc})}$ and ΔG_m for amino acid polymorphisms where the differences between variants were significant for both variables. Data is of IgG3 alleles only. Data for polymorphisms at position 435 is included, despite no statistical significance, to complement the analysis of collective polymorphisms at 435 and 436 together. Statistical significance was tested by one-way ANOVA with Tukey PostHoc multiple comparison testing. * $p < 0.05$, ** $p < 0.01$, *** $p < 0.001$, **** $p < 0.0001$.

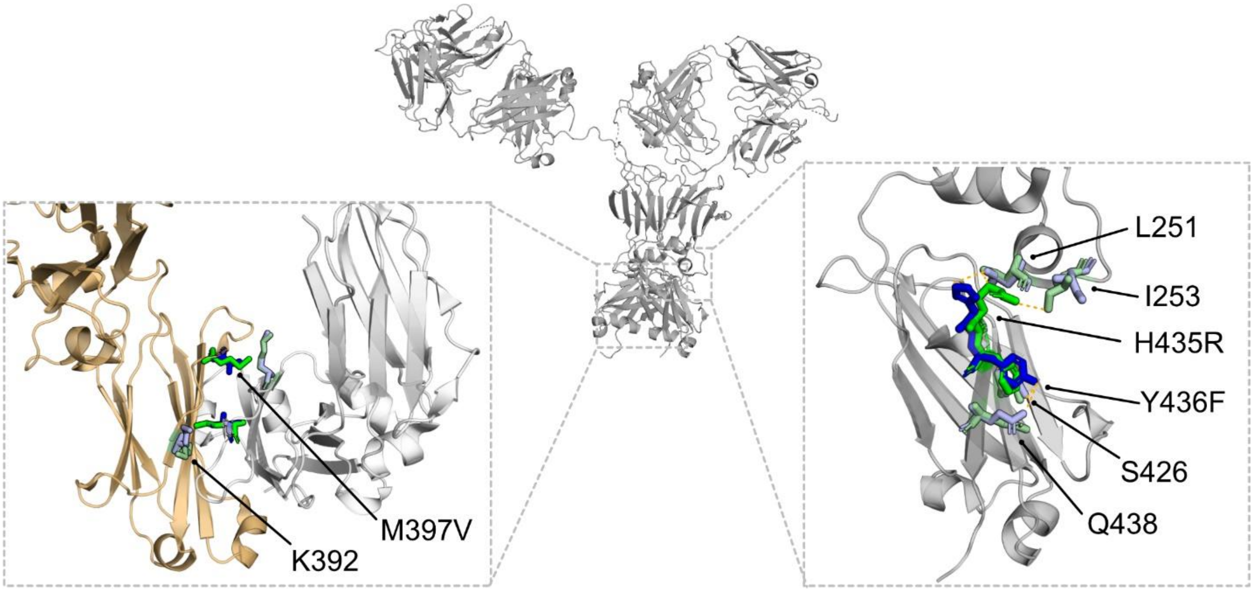

Figure S6. Structural alignment of amino acid mutations with significant differences in stability. Left panel: Met-397 (green) vs Val-397 (blue) at the interface of the CH3 domains. Lys-392 is also shown as this is suggested to have a role in stability in combination with 397. One half of the CH3 domain dimer is colored gold to aid the eye. Right panel: His-435 (blue) vs Arg-435 (green) and Tyr-436 (blue) vs Phe-436 (green). The interactions of key residues and surrounding side chains are indicated by the yellow dotted lines.

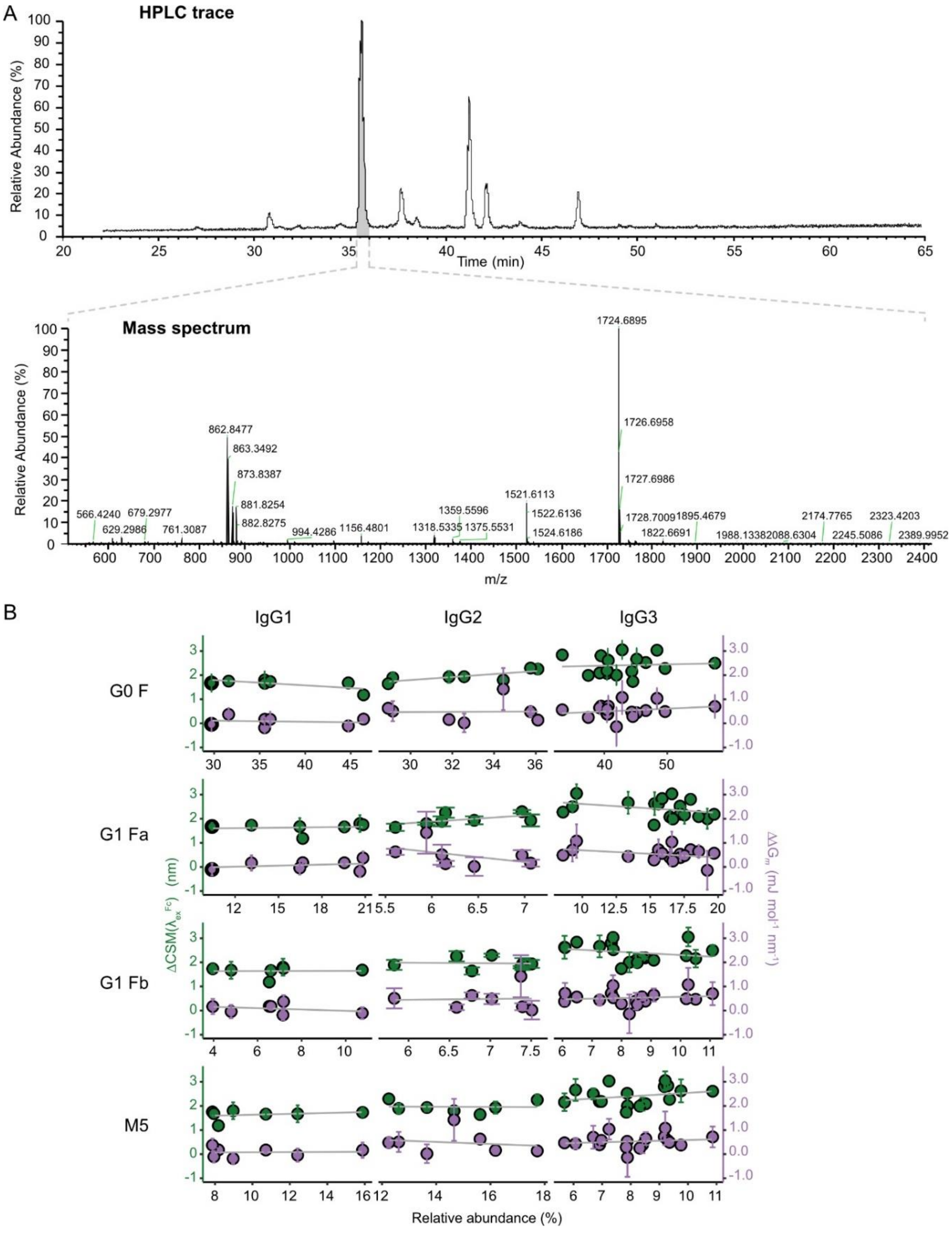

Figure S7 Effects of glycans on stability. (A) High-performance liquid chromatography chromatogram of free glycans released from antibody IgG1*07 and labelled with InstantPC dye. Example of mass spectrum for elution peak at approximately 35 min showing mass-to-charge ratios of glycoform, G0 F. (B) Correlation analysis of the abundance percentage of the four most abundant glycans with the change in $\boldsymbol{CSM(\lambda_{Ex}^{Fc})}$ (green, left y axis) and the change in ΔG_m (purple, right y axis) per subclass. Correlation coefficients and the p-value for each correlation are given in Table S4.
